# Comparison of different drying technologies for walnut (*Juglans regia* L.) pellicles: Changes from phenolic composition, antioxidant activity to potential application

**DOI:** 10.1016/j.fochx.2023.101037

**Published:** 2023-11-27

**Authors:** Li Qingyang, Wang Ruohui, Sun Shiman, Shen Danyu, Mo Runhong, Liu Yihua

**Affiliations:** Research Institute of Subtropical Forestry, Chinese Academy of Forestry, Fuyang 311400, PR China

**Keywords:** Walnut pellicle, Drying effect, Phenolic, Utilization, Active substances, KEGG

## Abstract

The analysis of the phenolic profile in the walnut pellicle (WP) and its exploitability can help to promote the valorization of the industrial waste from walnut production. Three forms of 33 monomeric phenols in WPs were quantified based on our previously established LC-MS/MS method. The levels of protocatechuic acid and 4-hydroxybenzoic acid in the WPs were the highest, exceeding 400 μg/g. Antioxidant tests revealed that all three phenolic forms of WPs were effective antioxidants (IC_50_: 2.12–35.05 µg/mL). The findings also revealed that drying temperature had a substantial type-dependent effect on phenolics and their antioxidant ability in WPs. KEGG enrichment analysis found that drying method has the greatest impact on WPs phenols in six metabolic pathways. Besides, 11 active substances in WPs were identified by a compound-targeted activity screening approach, indicating that WPs could be used as a natural antioxidant source in the development of medical and nutraceutical products.

## Highlights

• Phenolic profile and its form in walnut pellicle were quantified.
• Walnut pellicle can be utilized as a natural antioxidant source.
• The utilization of phenolics in pellicle is type dependent on drying temperature.
• Temperature sensitive phenolic and related metabolic mechanism was identified.

## 1 Introduction

In the last decade, there has been a growing interest in reusing and upgrading byproducts from plant processing industries. These industries, especially the food industry, produce a huge amount of solid and liquid waste, which creates serious environmental and health problems (Banožić et al., 2020). According to a study conducted by the Food and Agriculture Organization of the United Nations, one-third of the total food produced for human consumption (approximately 1300 million tons per year) is wasted worldwide (Arun et al., 2020). Food waste can be utilized as raw materials of various bioactive molecules, such as phenolics. Phenolics, including various phenolic acids and flavonoids, have gained increasing attention due to their potential health benefits for human beings. Phenolics are essential antioxidants linked to anti-carcinogenic, anti-inflammatory, antibacterial, anti-aging, anti-fungal, neuroprotective, and other functions (Ni et al., 2021). It should be noted that the phenolics market is expanding as demand grows in Europe, Japan, and North America due to an aging population and the anti-aging properties of these compounds (Malekjani & Jafari, 2022). In addition, consumers are turning to natural products for the treatment of some diseases, and the replacement of toxic artificial chemical preservatives in processed foods with safe products is increasing every day (Malekjani & Jafari, 2022). However, it is estimated that a large number of unknown, active compounds playing an important role in therapeutically treatment are yet to be discovered.

Walnut (*Juglans regia* L.) is an economic nut fruit valued for its abundant macronutrients, and is widely cultivated around the world. Walnut has the most diverse phenolic profile and the highest phenolic content among tree nuts (Persic et al., 2018). According to the performed studies, the skin or seed coat surrounding the kernel contains high amounts of phenolic compounds, resulting in the higher antioxidant activity of walnut kernel compared to other nuts (Jahanban-Esfahlan et al., 2019). According to some studies, the TPC content of the pellicle of walnut accounts for more than 90 % of the total walnut kernel (Jahanban-Esfahlan et al., 2019). However, the pellicle of walnut kernel is also an important waste product of the walnut processing industry. Therefore, the reuse of phenolics in the pellicles will be of great importance. In addition, nut pellicles have received considerable research attention as a significant reservoir of polyphenols in various nuts, including pistachios (Arcan & Yemenicioğlu, 2009) and hazelnuts (Persic, 2018). Existing studies, on the other hand, focuses on of total phenolic content, primarily by comparing kernel with and without pellicles. There are few comprehensive studies that specifically examine the polyphenol profiles of the nut pellicle in isolation.

In addition, previous works have shown that numerous structures of phenolics can be classified into three forms (free, esterified and bound), depending on their association with matrix (Lou et al., 2020). Most of the soluble phenolics are localized in the vacuoles of plant cells where they are trapped. Insoluble-bound phenolics are localized in cell wall matrix of the plant cells where they are bound via ether, ester and carbon–carbon bonds with macromolecules such as structural proteins, cellulose and pectin (Dibanda et al., 2020). Most of previous studies focused only on phenolic compounds in free fraction but did not examine bound phenolics in residues, thereby underestimating the actual content of walnuts (Wu et al., 2021). Moreover, the results showed that esterified and bound forms are rich in a variety of phenolics and can be an important source of natural antioxidant raw materials. Therefore, after the extraction of phenols in the free form of skin, further studies on phenols in the esterified and bound forms for their residues will help us to fully exploit the natural phenolic resources of pellicle.

Drying step is the key step in walnut industry. The process extends the shelf life of foods; yet, important quality features including color, taste and phenolic compounds should be preserved as much as possible for higher consumer preference (Harguindeguy and Fissore, 2020, Korkmaz et al., 2017). Different techniques are used for drying foods, including walnuts such as sun drying, hot air, and freeze drying (Liu et al., 2021, Pakrah et al., 2021). Traditional walnut drying methods include constant hot air-dried (CHD) and freeze-dried (FD) (Liu et al., 2021, Pakrah et al., 2021). Gradient hot air drying (GHD) has emerged as a promising alternative in recent years due to its superior efficiency. This method involves rapid dehydration at high temperatures followed by constant temperature drying, resulting in increased work efficiency. As a result, the purpose of this study was to evaluate the antioxidant capacity and phenolic compounds of walnuts using four different drying methods. We also used the Traditional Chinese Medicine System Pharmacology (TCMSP) database to look for active substances (ACSs) in walnuts. In addition, we established an ACS-target-disease network to investigate the potential industrial applications of walnut pellicles (WPs). Finally, we looked into the critical differential metabolic pathways and underlying mechanisms linked to these ACSs. The findings may support the valorization of industrial waste from walnut production.

## 2 Materials and methods

### 2.1 Chemicals and reagents

All targeted phenolic compounds (purity > 98 %), which were purchased from Sigma-Aldrich in Shanghai, China. HPLC grade methanol (MeOH), ethanol (EtOH), acetonitrile (ACN) and formic acid (FA) were purchased from Merck in Hangzhou, China.

### 2.2 Preparation of samples

The walnut germplasm resource nursery in Shanxi, China provided the samples. All samples (3 kg) were obtained from 10- to 15-year-old walnut trees at their commercial mature stage. At each collection location, we randomly collected 10 walnuts with the same degree of maturity from the same walnut tree. The detailed sampling method can be found in Wu et al (Wu et al., 2021). The nuts were manually cracked and shelled to obtain the walnut kernels.

To dry the walnuts, four drying methods were used: (1). Freeze drying (FD): Walnut kernels were spread in a single layer on trays and placed in a lyophilizer (FD-1D-80, China). The lyophilizer was set to a pressure of 30 Pa and a condenser temperature of −50 °C. (2). Constant hot air-dried (CHD): An electric thermo-static drying hot-air oven (XMA-2000, Germany) was set at 40 °C. (3). Gradient hot air-dried 1 (GHD1): Walnut kernels were dried at 80 °C for 4 h before being moved to 40 °C to dry further. (4). Gradient hot air-dried 2 (GHD2): Walnut kernels were dried at 105 °C for 2 h before being moved to 40 °C to dry further.

All drying methods were employed to reduce the moisture content of the walnuts below 8 %. The pellicles were manually separated from the kernels and stored at −20 °C for testing.

### 2.3 Extraction of phenolic compounds

The walnut pellicle (0.5 g) was extracted with 50 mL of 70 % acetone–water solution in a cold-water bath. The extraction process was repeated three times, and then the combined liquid extracts were flash-evaporated to remove the acetone. After the pH of the liquid extracts was adjusted using 2 M HCl to 2.0, liquid–liquid extraction was carried out using ethyl acetate (three times). The supernatants were evaporated and then re-dissolved in MS-grade methanol to obtain the free phenolics.

The leftover product and solid residue were simultaneously treated with 2 M NaOH for one hour in a nitrogen atmosphere. The sample was then acidified with 2 M HCl to pH 2.0 and phase separated with ethyl acetate. The subsequent operation of esterified and bound phenolics steps were consistent with those of the treatment process of the free phenolics. The details of the extraction process can be seen in our previous work (Wu et al., 2020).

### 2.4 Quantification of phenolic compound by UPLC-MS/MS

The ultra-performance liquid chromatography coupled with a triple quadrupole mass spectrometer (UPLC-MS/MS) consisted of an ultra-high performance liquid chromatography (Agilent 1290 Infinity II, Agilent Technologies Inc., California, USA) with Poroshell 120 EC-C18 column (100 × 2.1 mm, 1.9 μm), hyphenated to a triple quadrupole mass spectrometer (6460C QQQ, Agilent Technologies Inc., California, USA) equipped with electrospray ionization (ESI). Followed the method by Shen et al (Shen et al., 2021), the ESI analysis was performed both in positive and negative polarity using Dynamic Multiple Reaction Monitoring (DMRM) mode with a dwell time of 0.02 s. The mobile phase consisted of A (0.5 % formic acid in water) and B (acetonitrile and methanol in the ratio 8:2). The gradient conditions were as followed: 0–1 min/15 %–20 % B, 12.0 min/50 % B, 15.0 min/60 % B, 17.0–19.0 min/95 % B, 19.0–20.0 min/15 % B. The MS/MS system was operated using electrospray ionization (ESI) in the negative ion mode with a capillary voltage of 3.0 kV. The ionization source and desolation temperatures were programmed to 120 °C and 500 °C, respectively.

### 2.5 Determination of total phenolic compounds

The extraction and the analysis of TPC were performed on the walnut samples as described by Wang et al (Wang et al., 2022). 1 mL extracting solvent of phenolics (after dilution) mix with 0.5 mL of Folin-Ciocalteu reagent. 3 mL of sodium carbonate (Na2CO3, 75 g/L) was added after 5 min. Then, the final mixture was diluted to 10 mL and kept in the dark at 40 °C for 1 h and the TPCs were measured at 765 nm in a spectrophotometer (Perkin Elmer, USA).

### 2.6 Determination of antioxidant activity

Radical scavenging activities of 1,1-diphenyl-2-picrylhydrazylradical scavenging activity (DPPH) assay were measured as the protocol of Hazli et al (Mohd Hazli et al., 2019). IC_50_ was calculated as the concentration of each extract (mg/ml) needed to scavenge 50 % of the DPPH radicals with a lower IC_50_ indicating a higher antioxidant activity of a compound.

### 2.7 Identification of ACSs in WPs by bioinformatics analysis

The compounds with potential bioactive activity were searched for Traditional Chinese Medicine Database and Analysis Platform (TCMSP) with integrated criterion of oral bioavailability (OB) ≥ 25 % and druglike (DL) ≥ 0.07 (Gao et al., 2022).

### 2.8 Data analysis

Statistical significance of all data was determined by SPSS22.0. The orthogonal partial least-squares discriminant analysis (OPLS-DA) was used to discriminate phenolics profiles between different drying groups. The variable importance in the projection (VIP), *P*-value, and absolute Log2FC (foldchange) were used to determine the phenolics that were significantly regulated among the groups. The data visualization was performed using the Metware Cloud (https://cloud.metware.cn) and Origin2019b.

## 3 Results

### 3.1 TPC content in WP under different drying methods

**Fig. 1:**
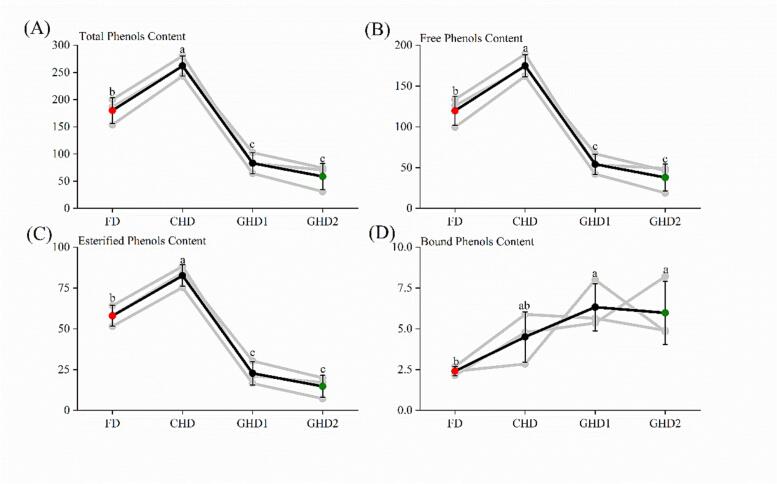
The content of TPC in WPs under different drying methods. Note: Different lowercase letters show significant difference (*p* < 0.05) in different drying methods. FD: Freeze drying; CHD: Constant hot air-dried; GHD1: Gradient hot air-dried 1; GHD2: Gradient hot air-dried 2.

The total phenolic content of three types (free, esterified, and bound) is shown in Fig. 1. The walnut pellicle under different drying conditions containing TPCs for free forms (37.89–174.77 mg/g), while bound and esterified forms contained TPCs in the range 14.70–82.63 mg/g and 2.40–6.33 mg/g, respectively. Under CHD, walnut pellicles exhibited the greatest total phenolic content at 261.90 mg/g, which was 1.46, 3.15, and 4.47 times greater than FD, GHD1, and GHD2 (Fig. 1A). The trend of free and esterified phenol content with different drying processes is consistent with that of total phenols, with CHD having the greatest amounts at 174.77 and 82.63 mg/g, respectively (Fig. 1B and C). The content of bound phenols in WPs rose generally linearly with the temperature. GHD had the largest level of bound phenols (average: 6.15 mg/g), which was 2.56 and 1.37 times greater than FD and CHD, respectively.

### 3.2 DPPH radical scavenging activity of WP under different drying methods

**Fig. 2:**
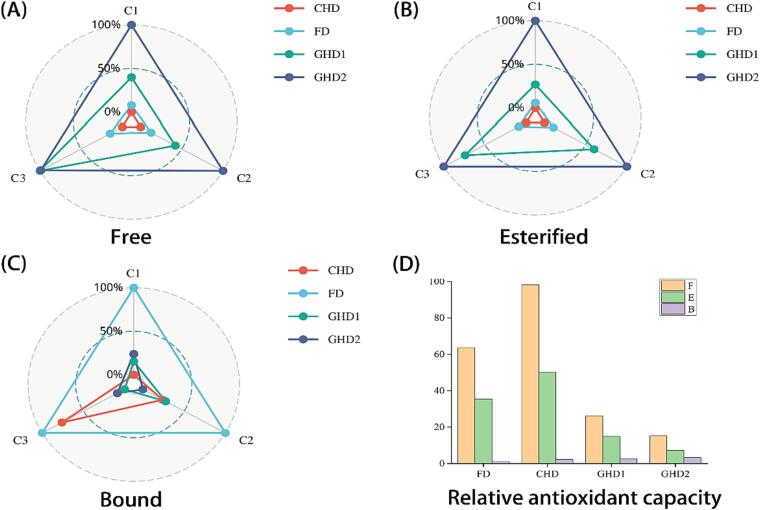
Anti-oxidant capacity of WPs under different drying methods. Note: Radar plot of anti-oxidant capacity on four drying methods assessed. The closer to the center of the radar plot, the higher the antioxidant capacity. FD: Freeze drying; CHD: Constant hot air-dried; GHD1: Gradient hot air-dried 1; GHD2: Gradient hot air-dried 2.

For free phenols, walnut pellicle had the strongest radical scavenging activity under CHD (average IC_50_ values: 0.67 µg/mL), followed by FD (1.03 µg/ mL), GHD1 (2.50 µg/ mL), and GHD2 (5.29 µg/mL) (Fig. 2A). The bound phenols were in the same order as the free phenols, with CHD having the highest free radical scavenging capacity (1.31 µg/mL) and GHD2 having the lowest (8.93 µg/mL) (Fig. 2B). Unlike free and esterified phenols, the ability of bound phenols to scavenge free radicals decreased linearly with increasing temperature. The free radical scavenging ability of bound phenols was 65.63, 30.99, 23.61 and 19.96 µg/mL for the four drying processes, respectively (Fig. 2C). The relative antioxidant capacity of the different forms of WPs was investigated further, and the results are presented in Fig. 2D. Free phenolics were shown to have the highest relative antioxidant capacity, up to 1.89 and 22.15 times that of esterified and bound phenolics, respectively.

### 3.3 The monomeric phenolic content of WP under different drying methods

**Fig. 3:**
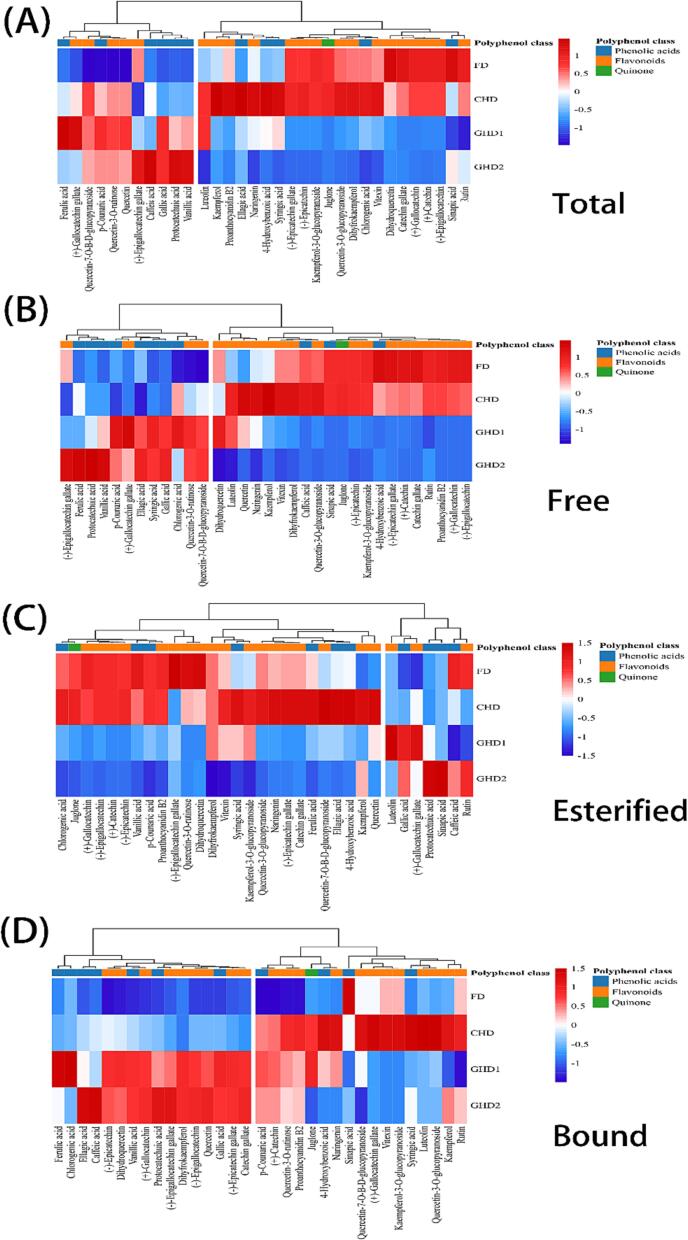
The monomeric phenolic content of WPs under different drying methods. Note: FD: Freeze drying; CHD: Constant hot air-dried; GHD1: Gradient hot air-dried 1; GHD2: Gradient hot air-dried 2.

We investigated the phenolic profiles in the pellicle following treatment with different drying temperatures using our previously described LC-MS/MS technique for monomeric phenols in walnut kernels (Shen et al., 2021). The four most prevalent monomeric phenols are protocatechuic acid, 4-hydroxybenzoic acid, ellagic acid, and gallic acid, with a total amount of more than 200 μg/g in all three forms. Heat maps were used to visualize variations in monomeric phenol content under different drying methods. The FD and CHD groups had the largest quantities of monomeric phenols identified in WPs, most notably (+)-catechin, catechin gallate, and (−)-epicatechin (Fig. 3A). This effect is particularly visible for free and esterified phenols (Fig. 3B and C). For free forms, almost all flavonoids arise in the group of FD and CHD. The FD group exhibited a notable dominance of four monomeric phenols, namely catechin gallate, (+)-catechin, (−)-epicatechin, and (−)-epicatechin gallate, with their abundance exceeding that of the GHD group by a factor of more than ten. The catechin gallate content of FD (52.16 μg/g) is even 60.55 times that of GHD (0.86 μg/g). The majority of the highest phenolic acid content values were observed in the GHD1 and GHD2 groups, with protocatechuic acid being the most remarkable. The greatest protocatechuic acid content in walnut pellicle under the GHD2 was 111.08, which was 49.60 times greater than in the FD and CHD.

These representative monomer phenols exhibit a similar trend in their esterified form as they do in their free form. In addition, because of its high content in CHD, up to 177.00 μg/g, which is 2.33 and 4.12 times greater than GHD1 and GHD2, respectively, ellagic acid must be addressed. CHD contains over half of the greatest monomeric phenol concentration for bound forms. It is also worth noting that the majority of these phenol contents are less than 1 μg/g, which is typical of sinapic acid, vitexin, and kaempferol-3-O-glucopyranoside. Higher content phenols, such as protocatechuic acid and gallic acid, were mostly discovered in the GHD group, with contents of 652.52 and 225.23 μg/g, respectively.

### 3.4 The identification of temperature-sensitive monomeric phenols in WPs

**Fig. 4:**
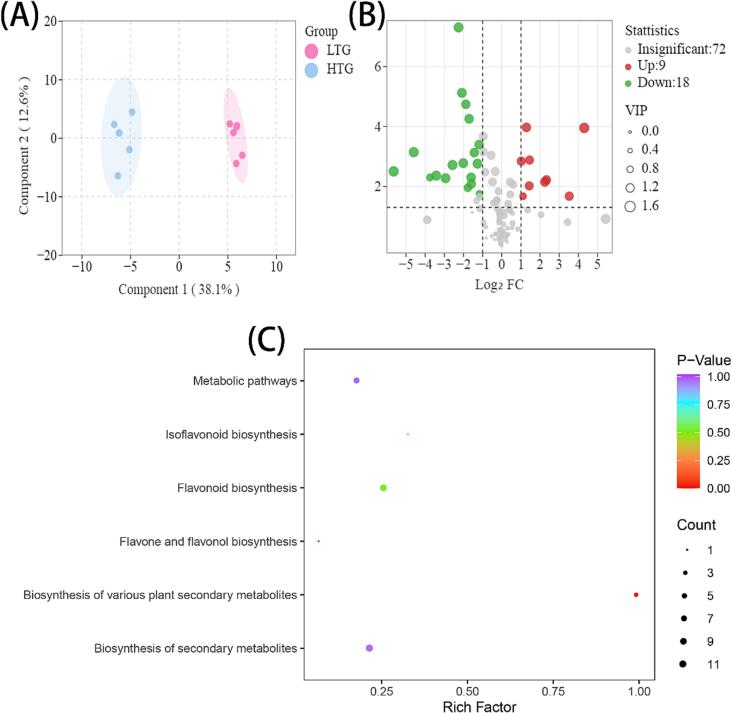
(A) OPLS-DA plot of monomeric phenolic between WPs under LTG and HTG group. (B) Volcano diagram of the differential phenolics of WPs under LTG and HTG group. (C) Metabolic pathway diagram for differences in LTG and HTG group. Note: For plot B: Red and green dots represent up-regulated and down-regulated significant (fold change ≥ 2 or fold change ≤ 0.5, p-value < 0.05, and VIP ≥ 1) difference phenolics, respectively. For plot C: The bubble size represents the number of different metabolites involved in this pathway, and the bubble color represents the p-value of this metabolic pathway. LTD: low temperature group; HTG: high temperature group. (For interpretation of the references to color in this figure legend, the reader is referred to the web version of this article.)

To better investigate the effect of temperature on the composition of different forms of phenols in walnut pellicle, we divided the temperature treatments into two groups (low temperature group (LTG) and high temperature group (HTG)) and used OPLS-DA to identify the structure of temperature-sensitive monomeric phenols and the corresponding forms. Fig. 4A shows that OPLS-DA can distinguish between the two treatment groups well, with 45 phenols variables regarded to have a significant contribution (VIP > 1) (Table S1). These phenols are mainly concentrated in the free (19) and esterified (16) forms, indicating that the bound phenols are largely temperature insensitivity. In addition, temperature-sensitive monomeric phenols and their forms are discovered using more stringent criteria such as *P* < 0.05, FC (Fold change) > 2 or < 0.5 and VIP ≥ 1. Under more stringent conditions, the levels of differential phenols between LTD and HTD were 27 (9 up and 18 down) to screen (Table S1 and Fig. 4B). According to our findings, phenolics can be categorized as follows depending on temperature sensitivity. Five monomeric phenols are considered highly sensitive, including catechin gallate, (−)-epicatechin, and (+)-catechin. The coefficient of variation (CV) of total catechin gallate content at different drying methods was up to 86.20 %. Ten monomeric phenols, including (−)-epigallocatechin and proanthocyanidin B2, were classified as moderately sensitive, with the CV ranging from 20 % to 65 % for the majority of them. The remaining monomeric phenols were classed as slightly sensitive since none of the three forms of these phenols were screened under the stringent screening conditions. Furthermore, the possible metabolic pathways of phenols under various drying conditions will be elucidated. The following pathways were identified as the most important: biosynthesis of various plant secondary metabolites, metabolic pathways, biosynthesis of secondary metabolites, flavonoid biosynthesis, flavone and flavonol biosynthesis, and isoflavonoid biosynthesis (Fig. 4C).

## 4 Discussions

### 4.1 Evaluation of WP as a source of natural antioxidant phenolic compounds

**Fig. 5:**
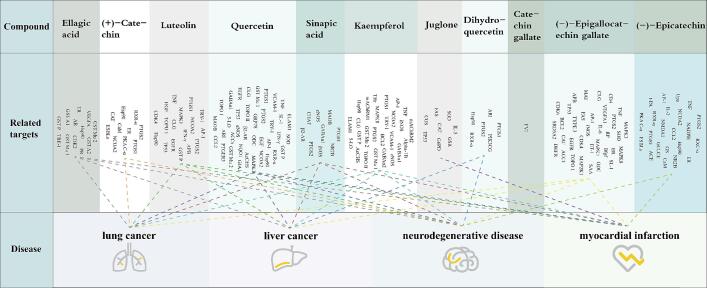
Target network and functional enrichment of the WPs.

The extraction of valuable phenolics from by-products can help to lessen the environmental impact of certain of these compounds while also providing a useful source of natural phenolics for the pharmaceutical and nutraceutical industries (Bhardwaj et al., 2022). In mostly previous works, many researchers used a variety of non-targeted activity screening method to investigate the function of phenolic chemicals in food processing by-products (Dibanda et al., 2020). Many intriguing new results have been revealed in recent years by a number of researchers who have built compound-targeted activity screening approaches based on a mix of databases and bioinformatics (Gao et al., 2023, Tao et al., 2022). In this study, we identified 11 ACSs as promising bioactive substances by scanning the TCMSP database for chemicals with OB ≥ 25 % and DL ≥ 0.07 (Table S2). The association between the associated possible bioactive compounds and their activity on prospective human health targets and diseases was then investigated (Table S3 and Table S4). The resulting “active substances”, “potential targets”, and “disease” were used to create a graph of compound-target-disease (C-*T*-D) interactions (Fig. 5).

These 11 ACSs are clearly possible target compounds for various targets, as well as potential medicines or nutraceuticals for a variety of neurological systems and malignancies. Among these 11 products, ellagic acid is the most prevalent monomeric phenol in the pellicle. This study revealed ellagic acid targets such as Glutathione S-transferase (GST) and Heat shock protein HSP 90 (Hsp90). These targets have gotten a lot of interest because of their importance in a number of clinical processes, such as liver cancer and neurodegenerative disorders (Jin et al., 2022, Lin et al., 2019). Animal studies have demonstrated that ellagic acid can reduce oxidative stress and hepatotoxicity caused by diclofenac (Gupta, et al., 2021a). Pomegranate juice, which is high in ellagic acid, has been proven to enhance verbal and visual memory deficiencies in older persons suffering from age-related memory loss (Bookheimer et al., 2013). Furthermore, the ellagic acid content of walnut pellicles (WPs) was found to be significantly higher (over 200 µg/g) than that of chestnuts (1.6–25 µg/g) and blackberries (15–22 µg/g), and comparable to that of strawberries (250–850 µg/g) (Gupta et al., 2021b). In light of previous research, our findings suggest that ellagic acid may have an effect on proteins linked to various diseases. As a result, the WPs have the potential to be a valuable resource in the development of medications or nutraceuticals that target neurological and other diseases.

The concentration of (+)-catechin in a study for apple peel utilization was found to be 20.50 μg/g (Gulsunoglu et al., 2019), which is regarded to have a favorable inhibitory impact on lung cancer. Catechin inhibited the proliferation of non-small cell lung cancer A549 cells by 19.76 % after 24 h of incubation at a concentration of 600 mu mol/L (174.17 μg /g) (Sun et al., 2020). (+)-Catechin, was found to have a significantly higher ACS concentration in WPs (>35 μg/g under different drying treatments)) than in apple peels. (+)-Catechin targets primarily include Prostaglandin-Endoperoxide Synthase (PTGS), which has received a lot of interest due to its important role in lung cancer (Lin et al., 2019). Previous study found that inhibition of PTGS1 and PTGS2 activity can effectively alleviate lung infections (Soni et al., 2021). This reveals walnut pellicle's significant potential for functional food creation or application in combination medicines for lung cancer treatment. In addition, our study discovered numerous ACSs, including quercetin, (−)-epigallocatechin gallate, and kaempferol, as well as over 40 targets related with them. This demonstrates their potential for application in a wide spectrum of diseases. Furthermore, our findings for pellicle antioxidant ability revealed that WP (IC_50_: free, 0.67–4.29 µg/mL; esterified: 1.85–8.93 µg/mL; bound: 19.96–65.63 µg/mL) has significantly higher antioxidant activity than pineapple peel (6500–15000 µg/mL) and orange pellicle (26200–38800 µg/mL), both of which are considered as processing residues with good natural antioxidant development value (Saleh et al., 2021). The majority of available research indicates that phenols' physiological and health effects are related to their antioxidant ability (Bhardwaj et al., 2022). As a result, our findings imply that the walnut pellicle has a high antioxidant ability, is rich in a variety of ACSs, and plays an important role in the treatment of many diseases such as liver and lung disorders. This emphasizes the importance of WPs as a natural antioxidant source for the development of medical and functional products.

### 4.2 Effect of postharvest drying method on ACSs in WP and related mechanism

**Fig. 6:**
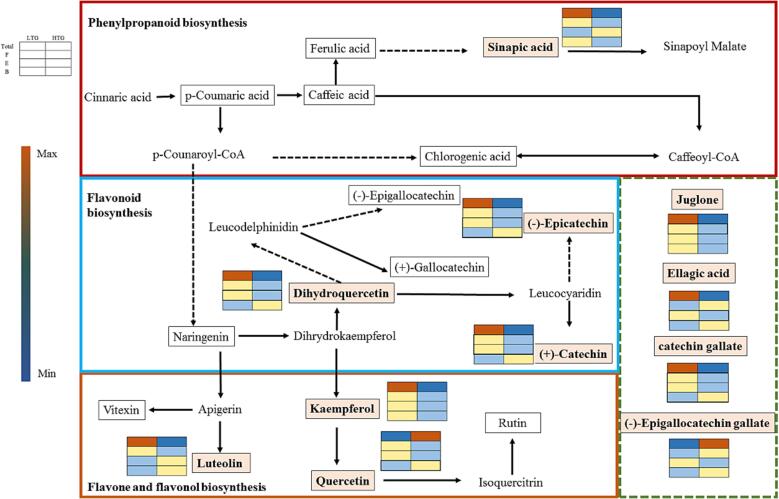
The network of ACS-related metabolic pathways. Note: The four ACS-associated metabolic pathways in the dashed box were not retrieved from the KEGG database.11 ACSs are labelled with pink undertones. The heat-map represented the relative content of the ACS between the two drying groups. (For interpretation of the references to color in this figure legend, the reader is referred to the web version of this article.)

Many researches have demonstrated that drying temperature has a substantial effect on the content and function of phenols in foods (Chumroenphat et al., 2021, Tan et al., 2021). Recent studies have also reported the benefits of drying for preserving various types and forms of phenols (Dibanda et al., 2020, Lang et al., 2019). However, there is little evidence on the relationship between temperature and different monomeric phenols, especially when combined with upstream and downstream metabolic pathway relationships to identify the appropriate metabolic mechanisms. In this study, we searched the KEGG database for the above mentioned 11 ACSs in WPs, and the ACS-related metabolic networks were presented (Fig. 6). The KEGG pathway enrichment analysis revealed that the ACSs were primarily involved in phenylpropanoid, flavonoids, and flavone and flavonol biosynthesis. Previous research has shown that these pathways result in the biosynthesis of flavonoids, isoflavones, and flavanols, all of which are important plant stress resistance phenolics (Ai et al., 2021). Drought stress and high temperature treatments, in particular, have been shown to induce the flavonoid biosynthetic pathway in response to stress (Chao et al., 2017, Yu et al., 2022). Surprisingly, the amounts of ACSs presented in these metabolic pathways were found to be mostly down-regulated in the HTG group. Under HTG conditions, the total content of (+)-catechin and (−)-epicatechin in flavonoid biosynthesis was only 0.26 and 0.24 times that of LTG, respectively. Furthermore, increasing the drying temperature from LTG to HTG reduced the TPCs of the two ACSs in the pathway of flavone and flavonol biosynthesis by 45.00 % (kaempferol) and 1.51 % (luteolin). This phenomenon could be caused by the effect of drying temperature on enzyme activity or substrate. This phenomenon was also discovered to be most pronounced in free and esterified phenols. When compared to the LTG group, the HTG group showed a decrease of over 90 % in the free form and more than 70 % in the esterified form of (+)-catechin and (−)-epicatechin. Overall, the free and esterified form content was primarily responsible for the decrease in TPCs and antioxidant ability of the 11 ACSs. During industrial processing, free and esterified phenols have been shown to exhibit higher extraction efficiency (Gulsunoglu et al., 2019). The distribution pattern of different forms of phenols under LTG treatment indicates that LTG treatment is better suited for direct extraction of these ACSs, allowing for their subsequent utilization.

In contrast, high temperatures have been shown to increase the content and antioxidant capacity of various phenols (not just ACSs). The bound form content of quercetin and (−)-epicatechin in the HTG group was found to be 2.70 and 2.16 times higher, respectively, than in the LTG group. Temperature has already been shown to favor the bound form of phenolic in black rice research, where drying at specific temperatures improves the complexation of free phenols with other components (Lang et al., 2019). As a result, more bound phenols are released at high temperatures. This temperature preference for the bound form has significant implications for phenol utilization. Bound phenolics, have been shown to survive digestion in the human stomach and small intestine and reach the colon intact, where they are released to exhibit their bioactivity with health benefits (Oboh & Ademosun, 2012).Therefore, for ACSs, particularly quercetin found in WPs, HTG treatment can be considered for its exploitation, because it can increase the content of the bound form, enhancing its effectiveness in the human body as a medicine or nutraceutical.

## 5 Conclusion

Overall, this is the first study to explore the phenolic profile and its form in WP, as well as to assess the possible utilization of industrial waste from walnut production. Our results discovered WP, as by-products of walnut industry, can be utilized as a natural antioxidant source in the development of medical and nutraceutical products. The drying temperature has a significant influence on the subsequent exploitation of WP as a natural antioxidant. Low-temperature drying is advised for industrial extraction of phenolics from WPs. High-temperature drying is recommended for extracting more active substances in the bound form or for the utilization of raw materials in the bound form. The utilized of WP through the extraction of its bioactive compounds may be a good solution to the environmental pollution and resource waste produced by the walnut industry.

## CRediT authorship contribution statement

**Li Qingyang:** Writing – original draft, Conceptualization, Visualization, Investigation, Data curation. **Wang Ruohui:** Investigation, Validation, Methodology. **Sun Shiman:** Visualization, Formal analysis. **Shen Danyu:** Methodology, Validation. **Mo Runhong:** Investigation. **Liu Yihua:** Writing – review & editing, Conceptualization, Software, Visualization.

## Declaration of competing interest

The authors declare that they have no known competing financial interests or personal relationships that could have appeared to influence the work reported in this paper.

## Data Availability

Data will be made available on request.
