# Supplementary material for: Comparison of different drying technologies for walnut (Juglans regia L.) pellicles: Changes from phenolic composition, antioxidant activity to potential application
Source: Food Chem X. 2023 Nov 27;20:101037. doi: 10.1016/j.fochx.2023.101037 (PMC10739750; doi:10.1016/j.fochx.2023.101037)
Supplement: Supplementary data 1 [file mmc1.docx]

**Supplementary material**

**Table S1 Heat sensitivity classification of 33 monomer phenols**

| Polyphenol class | Phenol | F | E | B |
| --- | --- | --- | --- | --- |
| Phenolic acids | Ellagic acid | <1 | 1.04 | <1 |
| Phenolic acids | Protocatechuic acid | 1.52 | 1.37 | 1.35 |
| Phenolic acids | 4-Hydroxybenzoic acid | <1 | <1 | <1 |
| Phenolic acids | Caffeic acid | <1 | <1 | <1 |
| Phenolic acids | Vanillic acid | 1.05 | <1 | <1 |
| Phenolic acids | Syringic acid | 1.08 | <1 | <1 |
| Phenolic acids | Gallic acid | 1.61 | 1.38 | 1.43 |
| Phenolic acids | Ferulic acid | <1 | <1 | <1 |
| Phenolic acids | Chlorogenic acid | <1 | 1.35 | <1 |
| Phenolic acids | p-Counaric acid | 1.13 | <1 | <1 |
| Phenolic acids | Sinapic acid | <1 | <1 | 1.02 |
| [Flavonoids](http://phenol-explorer.eu/classifications/compounds/1) | (+)-Catechin | 1.56 | 1.42 | <1 |
| [Flavonoids](http://phenol-explorer.eu/classifications/compounds/1) | (-)-Epicatechin gallate | 1.54 | 1.43 | 1.37 |
| [Flavonoids](http://phenol-explorer.eu/classifications/compounds/1) | (+)-Gallocatechin | 1.51 | 1.34 | 1.24 |
| [Flavonoids](http://phenol-explorer.eu/classifications/compounds/1) | (-)-Epigallocatechin | 1.55 | 1.38 | <1 |
| [Flavonoids](http://phenol-explorer.eu/classifications/compounds/1) | (-)-Epigallocatechin gallate | <1 | <1 | <1 |
| [Flavonoids](http://phenol-explorer.eu/classifications/compounds/1) | (-)-Epicatechin | 1.25 | 1.53 | 1.06 |
| [Flavonoids](http://phenol-explorer.eu/classifications/compounds/1) | Catechin gallate | 1.59 | 1.43 | 1.46 |
| [Flavonoids](http://phenol-explorer.eu/classifications/compounds/1) | (+)-Gallocatechin gallate | <1 | <1 | <1 |
| [Flavonoids](http://phenol-explorer.eu/classifications/compounds/1) | Vitexin | 1.33 | 1.11 | 1.04 |
| [Flavonoids](http://phenol-explorer.eu/classifications/compounds/1) | Luteolin | <1 | <1 | <1 |
| [Flavonoids](http://phenol-explorer.eu/classifications/compounds/1) | Kaempferol | <1 | <1 | <1 |
| [Flavonoids](http://phenol-explorer.eu/classifications/compounds/1) | Quercetin-3-O-rutinose | <1 | <1 | <1 |
| [Flavonoids](http://phenol-explorer.eu/classifications/compounds/1) | Rutin | 1.16 | <1 | <1 |
| [Flavonoids](http://phenol-explorer.eu/classifications/compounds/1) | Quercetin-7-O-B-D-glucopyranoside | <1 | <1 | <1 |
| [Flavonoids](http://phenol-explorer.eu/classifications/compounds/1) | Quercetin-3-O-glucopyranoside | 1.50 | 1.47 | <1 |
| [Flavonoids](http://phenol-explorer.eu/classifications/compounds/1) | Dihyfrokaempferol | 1.48 | <1 | <1 |
| [Flavonoids](http://phenol-explorer.eu/classifications/compounds/1) | Dihydroquercetin | <1 | 1.26 | 1.17 |
| [Flavonoids](http://phenol-explorer.eu/classifications/compounds/1) | Quercetin | <1 | <1 | 1.35 |
| [Flavonoids](http://phenol-explorer.eu/classifications/compounds/1) | Naringenin | 1.02 | 1.10 | <1 |
| [Flavonoids](http://phenol-explorer.eu/classifications/compounds/1) | Kaempferol-3-O-glucopyranoside | 1.44 | <1 | <1 |
| [Flavonoids](http://phenol-explorer.eu/classifications/compounds/1) | Proanthocyanidin B2 | 1.51 | 1.26 | <1 |
| Quinone | Juglone | 1.51 | 1.29 | <1 |

Note: Monomer phenols with high heat sensitivity, medium heat sensitivity and sight heat sensitivity are marked with orange, yellow and blue background colors, respectively. The data displayed in columns F, E, B represent the VIP values, and those that simultaneously satisfy the stringent criteria of *P* < 0.05, FC (Fold change) > 2 or < 0.5 and VIP ≥ 1 are marked with a green background color.

**Table S2 11 compounds with potential activity from WPs**

| No | Compounds | Classification | Total content (µg/g) | CAS | OB | DL |
| --- | --- | --- | --- | --- | --- | --- |
| 1 | Ellagic acid | Phenolic acids | 208.78-290.46 | 476-66-4 | 43.06 | 0.43 |
| 2 | (+)-Catechin | Flavonoids | 36.76-159.24 | 154-23-4 | 54.83 | 0.24 |
| 3 | Catechin gallate | Flavonoids | 8.35-55.88 | 1257-08-5 | 53.57 | 0.75 |
| 4 | (-)-Epicatechin | Flavonoids | 7.88-41.93 | 2545-08-6 | 28.93 | 0.24 |
| 5 | Dihydroquercetin | Flavonoids | 7.60-27.26 | 480-18-2 | 57.84 | 0.27 |
| 6 | Quercetin | Flavonoids | 5.95-11.77 | 73123-10-1 | 46.43 | 0.28 |
| 7 | (-)-Epigallocatechin gallate | Flavonoids | 4.18-4.52 | 989-51-5 | 55.09 | 0.77 |
| 8 | Juglone | Quinone | 1.54-5.73 | 481-39-0 | 25.74 | 0.07 |
| 9 | Kaempferol | Flavonoids | 2.14-5.30 | 520-18-3 | 41.88 | 0.24 |
| 10 | Luteolin | Flavonoids | 1.91-2.05 | 491-70-3 | 36.16 | 0.25 |
| 11 | Sinapic acid | Phenolic acids | 1.25-1.41 | 530-59-6 | 64.15 | 0.08 |

**Table S3 Related diseases of 11 active substances**

| Compound | Related diseases |
| --- | --- |
| (-)-Epicatechin | Osteoarthritis |
| (-)-Epicatechin | Malignant mesothelioma |
| (-)-Epicatechin | Lung Cancer |
| (-)-Epicatechin | Oropharyngeal squamous cell carcinoma |
| (-)-Epicatechin | Meningioma |
| (-)-Epicatechin | Pathological angiogenesis |
| (-)-Epicatechin | Peutz-Jeghers syndrome |
| (-)-Epicatechin | Prostate cancer |
| (-)-Epicatechin | Pyresis |
| (-)-Epicatechin | Renal Cell Carcinoma |
| (-)-Epicatechin | Rheumatoid arthritis, unspecified |
| (-)-Epicatechin | Pain, unspecified |
| (-)-Epicatechin | Cardiovascular disease, unspecified |
| (-)-Epicatechin | Migraine |
| (-)-Epicatechin | Dysmenorrhea, unspecified |
| (-)-Epicatechin | Chronic inflammatory diseases |
| (-)-Epicatechin | Carpal tunnel syndrome |
| (-)-Epicatechin | Endocrine independent cancer |
| (-)-Epicatechin | Analgesics |
| (-)-Epicatechin | Gestational hypertension |
| (-)-Epicatechin | Genitourinary tumors |
| (-)-Epicatechin | Brain injury |
| (-)-Epicatechin | Coronary atherosclerosis |
| (-)-Epicatechin | Postmenopausal symptoms |
| (-)-Epicatechin | Osteoporosis |
| (-)-Epicatechin | Cardiovascular disease |
| (-)-Epicatechin | Abdominal aortic aneurysm |
| (-)-Epicatechin | Alzheimer's Disease |
| (-)-Epicatechin | Bladder cancer |
| (-)-Epicatechin | Cancer, unspecific |
| (-)-Epicatechin | Carcinoma in situ, unspecified |
| (-)-Epicatechin | Colorectal cancer |
| (-)-Epicatechin | Endometriosis |
| (-)-Epicatechin | Inflammatory diseases |
| (-)-Epicatechin | Myocardial Infarction |
| (-)-Epicatechin | Neurodegenerative diseases |
| (-)-Epicatechin | Osteoporosis, unspecified |
| (-)-Epicatechin | Breast cancer |
| (-)-Epicatechin | Arthritis |
| (-)-Epicatechin | Adenomatous polyposis |
| (-)-Epicatechin | Inflammation |
| (-)-Epicatechin | Vascular disease |
| (-)-Epicatechin | Chronic obstructive pulmonary disease, unspecified |
| (-)-Epicatechin | Ovarian cancer |
| (-)-Epicatechin | Solid tumors |
| (-)-Epicatechin | Congestive Heart Failure |
| (-)-Epicatechin | Not Available |
| (-)-Epicatechin | Noninsulin-dependent diabetes mellitus |
| (-)-Epicatechin | Hypertension |
| (-)-Epicatechin | Gastrointestinal Stromal Tumors (GIST) |
| (-)-Epicatechin | Stroke |
| (-)-Epicatechin | Pain, unspecific |
| (-)-Epicatechin | Bacterial Infections |
| (-)-Epicatechin | HER2-positive Metastatic Breast Cancer |
| (-)-Epicatechin | Chronic Myelogenous Leukemia (CML) |
| (-)-Epicatechin | Melanoma |
| (-)-Epicatechin | Hyperimmunoglobulinemia D |
| (-)-Epicatechin | Crohns's Disease, unspecified |
| (-)-Epicatechin | Renal ischemia-reperfusion injury |
| (-)-Epicatechin | Hematological Malignancies |
| (-)-Epicatechin | Rheumatic diseases |
| (-)-Epicatechin | Cancer (multidrug resistant) |
| (-)-Epicatechin | Refractory Hematological Malignancies |
| (-)-Epicatechin | Multiple Myeloma |
| (-)-Epicatechin | Asthma |
| (-)-Epicatechin | Heart Failure |
| (-)-Epicatechin | Allergic airway inflammation |
| (-)-Epicatechin | Rheumatoid arthritis |
| (-)-Epicatechin | Cachexia |
| (-)-Epicatechin | Hyperinflammatory provoked organ injury |
| (-)-Epicatechin | Inflammatory Disorders, Unspecified |
| (-)-Epicatechin | Systemic-onset juvenile idiopathic arthritis |
| (-)-Epicatechin | Kidney Transplantation |
| (-)-Epicatechin | Acute coronary syndromes |
| (-)-Epicatechin | Atherosclerosis |
| (-)-Epicatechin | Peyronie's disease |
| (-)-Epicatechin | Neurological diseases |
| (-)-Epicatechin | Behcet's disease |
| (-)-Epicatechin | Glioblastoma multiforme |
| (-)-Epicatechin | Vascular lesion regression |
| (-)-Epicatechin | Solid Tumor |
| (-)-Epicatechin | Non-small Cell Lung Cancer |
| (-)-Epicatechin | Periodic fever syndrome |
| (-)-Epicatechin | Inflammatory diseases associated with hypoxia |
| (-)-Epigallocatechin gallate | Squamous cell carcinoma |
| (-)-Epigallocatechin gallate | Tumors |
| (-)-Epigallocatechin gallate | Colorectal Neoplasms |
| (-)-Epigallocatechin gallate | Angiogenesis |
| (-)-Epigallocatechin gallate | Lymphangiomatosis |
| (-)-Epigallocatechin gallate | Solid Tumor |
| (-)-Epigallocatechin gallate | Glioblastoma multiforme |
| (-)-Epigallocatechin gallate | Cancer, unspecific |
| (-)-Epigallocatechin gallate | Ovarian cancer |
| (-)-Epigallocatechin gallate | Macular Degeneration |
| (-)-Epigallocatechin gallate | Peripheral Vascular Disease |
| (-)-Epigallocatechin gallate | Solid tumors |
| (-)-Epigallocatechin gallate | Ulcers |
| (-)-Epigallocatechin gallate | Severe Coronary Heart Disease |
| (-)-Epigallocatechin gallate | Prostate cancer (hormone refractory) |
| (-)-Epigallocatechin gallate | Inflammatory diseases |
| (-)-Epigallocatechin gallate | Hepatocellular carcinoma |
| (-)-Epigallocatechin gallate | Hormone-refractory Prostate cancer |
| (-)-Epigallocatechin gallate | Lung Cancer |
| (-)-Epigallocatechin gallate | Prostate cancer |
| (-)-Epigallocatechin gallate | Non-small Cell Lung Cancer |
| (-)-Epigallocatechin gallate | Waldenstrom's macroglobulinemia |
| (-)-Epigallocatechin gallate | HCV infection |
| (-)-Epigallocatechin gallate | Brain Cancer |
| (-)-Epigallocatechin gallate | Melanoma |
| (-)-Epigallocatechin gallate | Osteoarthritis |
| (-)-Epigallocatechin gallate | Angiogenesis in metastatic and atherosclerotic processes |
| (-)-Epigallocatechin gallate | Colorectal cancer |
| (-)-Epigallocatechin gallate | Chronic lymphocytic leukemia |
| (-)-Epigallocatechin gallate | Pancreatic Cancer |
| (-)-Epigallocatechin gallate | Head and neck tumors |
| (-)-Epigallocatechin gallate | Kaposi's Sarcoma |
| (-)-Epigallocatechin gallate | Breast cancer |
| (-)-Epigallocatechin gallate | Renal Cell Carcinoma |
| (-)-Epigallocatechin gallate | Anemia due to disorders of glutathione metabolism |
| (-)-Epigallocatechin gallate | lschemic injury of the liver |
| (-)-Epigallocatechin gallate | Other phagocyte defects |
| (-)-Epigallocatechin gallate | Dementia |
| (-)-Epigallocatechin gallate | Chronic Renal Failure |
| (-)-Epigallocatechin gallate | Gout |
| (-)-Epigallocatechin gallate | Hereditary Polyposis Syndromes |
| (-)-Epigallocatechin gallate | Precursor Cell Lymphoblastic Leukemia-Lymphoma |
| (-)-Epigallocatechin gallate | Leukemia, Unspecified |
| (-)-Epigallocatechin gallate | Sleeping Sickness |
| (-)-Epigallocatechin gallate | Metastatic osteosarcoma in the lung |
| (-)-Epigallocatechin gallate | Emphysema |
| (-)-Epigallocatechin gallate | African trypanosomiasis |
| (-)-Epigallocatechin gallate | Hypertension |
| (-)-Epigallocatechin gallate | Chondrosarcoma |
| (-)-Epigallocatechin gallate | Mesothelioma |
| (-)-Epigallocatechin gallate | Benign prostate hyperplasia |
| (-)-Epigallocatechin gallate | Endometrial carcinoma |
| (-)-Epigallocatechin gallate | Osteoporosis |
| (-)-Epigallocatechin gallate | Migraine |
| (-)-Epigallocatechin gallate | Cardiovascular disease |
| (-)-Epigallocatechin gallate | Bacterial Infections |
| (-)-Epigallocatechin gallate | Alopecia, unspecified |
| (-)-Epigallocatechin gallate | Androgen-dependent diseases |
| (-)-Epigallocatechin gallate | Atopic asthma |
| (-)-Epigallocatechin gallate | Brain injury |
| (-)-Epigallocatechin gallate | Coronary atherosclerosis |
| (-)-Epigallocatechin gallate | Parkinson's disease |
| (-)-Epigallocatechin gallate | Endocrine independent cancer |
| (-)-Epigallocatechin gallate | Postmenopausal symptoms |
| (-)-Epigallocatechin gallate | LPS-induced left ventricular dysfunction |
| (-)-Epigallocatechin gallate | Malaria |
| (-)-Epigallocatechin gallate | Hepatic fibrosis |
| (-)-Epigallocatechin gallate | Cardiovascular disease, unspecified |
| (-)-Epigallocatechin gallate | Kidney Cancer |
| (-)-Epigallocatechin gallate | Hyperimmunoglobulinemia D |
| (-)-Epigallocatechin gallate | Inflammatory Disorders, Unspecified |
| (-)-Epigallocatechin gallate | Rectal Neoplasms |
| (-)-Epigallocatechin gallate | Multiple Myeloma |
| (-)-Epigallocatechin gallate | Cachexia |
| (-)-Epigallocatechin gallate | Proliferative diseases |
| (-)-Epigallocatechin gallate | Smooth muscle hyperplasia |
| (-)-Epigallocatechin gallate | Chronic inflammatory diseases |
| (-)-Epigallocatechin gallate | Insulin-dependent diabetes mellitus |
| (-)-Epigallocatechin gallate | Asthma |
| (-)-Epigallocatechin gallate | Rheumatoid arthritis |
| (-)-Epigallocatechin gallate | Congestive Heart Failure |
| (-)-Epigallocatechin gallate | Heart Failure |
| (-)-Epigallocatechin gallate | Noninsulin-dependent diabetes mellitus |
| (-)-Epigallocatechin gallate | Periodic fever syndrome |
| (-)-Epigallocatechin gallate | Rheumatic diseases |
| (-)-Epigallocatechin gallate | Rheumatoid arthritis, unspecified |
| (-)-Epigallocatechin gallate | Cancer (multidrug resistant) |
| (-)-Epigallocatechin gallate | Vascular disease |
| (-)-Epigallocatechin gallate | Obesity |
| (-)-Epigallocatechin gallate | Systemic-onset juvenile idiopathic arthritis |
| (-)-Epigallocatechin gallate | Osteoporosis, unspecified |
| (-)-Epigallocatechin gallate | Bladder cancer |
| (-)-Epigallocatechin gallate | Hearing Loss |
| (-)-Epigallocatechin gallate | Endometriosis |
| (-)-Epigallocatechin gallate | Abdominal aortic aneurysm |
| (-)-Epigallocatechin gallate | Adenomatous polyposis |
| (-)-Epigallocatechin gallate | Alzheimer's Disease |
| (-)-Epigallocatechin gallate | Carcinoma in situ, unspecified |
| (-)-Epigallocatechin gallate | Carpal tunnel syndrome |
| (-)-Epigallocatechin gallate | Dysmenorrhea, unspecified |
| (-)-Epigallocatechin gallate | Inflammation |
| (-)-Epigallocatechin gallate | Genitourinary tumors |
| (-)-Epigallocatechin gallate | Gestational hypertension |
| (-)-Epigallocatechin gallate | Malignant mesothelioma |
| (-)-Epigallocatechin gallate | Meningioma |
| (-)-Epigallocatechin gallate | Pathological angiogenesis |
| (-)-Epigallocatechin gallate | Oropharyngeal squamous cell carcinoma |
| (-)-Epigallocatechin gallate | Pain, unspecified |
| (-)-Epigallocatechin gallate | Peutz-Jeghers syndrome |
| (-)-Epigallocatechin gallate | Pyresis |
| (-)-Epigallocatechin gallate | Stroke |
| (-)-Epigallocatechin gallate | Vascular lesion regression |
| (-)-Epigallocatechin gallate | Kidney Transplantation |
| (-)-Epigallocatechin gallate | Analgesics |
| (-)-Epigallocatechin gallate | Traumatic brain injury |
| (-)-Epigallocatechin gallate | Myocardial Infarction |
| (-)-Epigallocatechin gallate | Crohns's Disease, unspecified |
| (-)-Epigallocatechin gallate | Myocardial infarction (MI) |
| (-)-Epigallocatechin gallate | Behcet's disease |
| (-)-Epigallocatechin gallate | Ischemia reperfusion injuries |
| (-)-Epigallocatechin gallate | Hyperinflammatory provoked organ injury |
| (-)-Epigallocatechin gallate | Arthritis |
| (-)-Epigallocatechin gallate | Neurodegenerative diseases |
| (-)-Epigallocatechin gallate | Insulin resistance |
| (-)-Epigallocatechin gallate | Allergic airway inflammation |
| (+)-Catechin | Stroke |
| (+)-Catechin | Inflammatory diseases |
| (+)-Catechin | Breast cancer |
| (+)-Catechin | Gestational hypertension |
| (+)-Catechin | Cardiovascular disease, unspecified |
| (+)-Catechin | Chronic inflammatory diseases |
| (+)-Catechin | Endocrine independent cancer |
| (+)-Catechin | Neurodegenerative diseases |
| (+)-Catechin | Osteoporosis, unspecified |
| (+)-Catechin | Postmenopausal symptoms |
| (+)-Catechin | Osteoporosis |
| (+)-Catechin | Myocardial Infarction |
| (+)-Catechin | Migraine |
| (+)-Catechin | Cardiovascular disease |
| (+)-Catechin | Abdominal aortic aneurysm |
| (+)-Catechin | Adenomatous polyposis |
| (+)-Catechin | Alzheimer's Disease |
| (+)-Catechin | Arthritis |
| (+)-Catechin | Bladder cancer |
| (+)-Catechin | Colorectal cancer |
| (+)-Catechin | Dysmenorrhea, unspecified |
| (+)-Catechin | Genitourinary tumors |
| (+)-Catechin | Inflammation |
| (+)-Catechin | Malignant mesothelioma |
| (+)-Catechin | Oropharyngeal squamous cell carcinoma |
| (+)-Catechin | Osteoarthritis |
| (+)-Catechin | Pain, unspecified |
| (+)-Catechin | Pathological angiogenesis |
| (+)-Catechin | Peutz-Jeghers syndrome |
| (+)-Catechin | Renal Cell Carcinoma |
| (+)-Catechin | Rheumatoid arthritis, unspecified |
| (+)-Catechin | Chronic Myelogenous Leukemia (CML) |
| (+)-Catechin | HER2-positive Metastatic Breast Cancer |
| (+)-Catechin | Multiple Myeloma |
| (+)-Catechin | Non-small Cell Lung Cancer |
| (+)-Catechin | Ovarian cancer |
| (+)-Catechin | Refractory Hematological Malignancies |
| (+)-Catechin | Solid tumors |
| (+)-Catechin | Bacterial Infections |
| (+)-Catechin | Not Available |
| (+)-Catechin | Heart Failure |
| (+)-Catechin | Precursor Cell Lymphoblastic Leukemia-Lymphoma |
| (+)-Catechin | Gastrointestinal Stromal Tumors (GIST) |
| (+)-Catechin | Cancer, unspecific |
| (+)-Catechin | Brain injury |
| (+)-Catechin | Pyresis |
| (+)-Catechin | Meningioma |
| (+)-Catechin | Hematological Malignancies |
| (+)-Catechin | Prostate cancer |
| (+)-Catechin | Carcinoma in situ, unspecified |
| (+)-Catechin | Carpal tunnel syndrome |
| (+)-Catechin | Lung Cancer |
| (+)-Catechin | Coronary atherosclerosis |
| (+)-Catechin | Analgesics |
| (+)-Catechin | Vascular lesion regression |
| (+)-Catechin | Endometriosis |
| (+)-Catechin | Melanoma |
| Catechin gallate | Coagulative disorders |
| Dihydroquercetin | Cardiovascular disease, unspecifed |
| Dihydroquercetin | Chronic inflammatory diseases |
| Dihydroquercetin | Abdominal aortic aneurysm |
| Dihydroquercetin | Adenomatous polyposis |
| Dihydroquercetin | Alzheimer's Disease |
| Dihydroquercetin | Analgesics |
| Dihydroquercetin | Arthritis |
| Dihydroquercetin | Bladder cancer |
| Dihydroquercetin | Breast cancer |
| Dihydroquercetin | Cancer, unspecific |
| Dihydroquercetin | Carcinoma in situ, unspecified |
| Dihydroquercetin | Carpal tunnel syndrome |
| Dihydroquercetin | Colorectal cancer |
| Dihydroquercetin | Dysmenorrhea, unspecified |
| Dihydroquercetin | Endometriosis |
| Dihydroquercetin | Genitourinary tumors |
| Dihydroquercetin | Gestational hypertension |
| Dihydroquercetin | Inflammation |
| Dihydroquercetin | Inflammatory diseases |
| Dihydroquercetin | Lung Cancer |
| Dihydroquercetin | Malignant mesothelioma |
| Dihydroquercetin | Meningioma |
| Dihydroquercetin | Myocardial Infarction |
| Dihydroquercetin | Oropharyngeal squamous cell carcinoma |
| Dihydroquercetin | Osteoarthritis |
| Dihydroquercetin | Pain, unspecified |
| Dihydroquercetin | Pathological angiogenesis |
| Dihydroquercetin | Peutz-Jeghers syndrome |
| Dihydroquercetin | Prostate cancer |
| Dihydroquercetin | Pyresis |
| Dihydroquercetin | Renal Cell Carcinoma |
| Dihydroquercetin | Rheumatoid arthritis, unspecified |
| Dihydroquercetin | Stroke |
| Dihydroquercetin | Vascular lesion regression |
| Dihydroquercetin | Chronic Myelogenous Leukemia (CML) |
| Dihydroquercetin | Gastrointestinal Stromal Tumors (GIST) |
| Dihydroquercetin | Hematological Malignancies |
| Dihydroquercetin | HER2-positive Metastatic Breast Cancer |
| Dihydroquercetin | Melanoma |
| Dihydroquercetin | Multiple Myeloma |
| Dihydroquercetin | Non-small Cell Lung Cancer |
| Dihydroquercetin | Ovarian cancer |
| Dihydroquercetin | Refractory Hematological Malignancies |
| Dihydroquercetin | Solid tumors |
| Dihydroquercetin | Angioedema |
| Dihydroquercetin | Heart Failure |
| Dihydroquercetin | Diabetic complications |
| Dihydroquercetin | Diabetic neuropathy |
| Dihydroquercetin | Diabetic retinopathy |
| Dihydroquercetin | Neuropathic pain |
| Dihydroquercetin | Noninsulin-dependent diabetes mellitus |
| Ellagic acid | Precursor Cell Lymphoblastic L eukemia-Lymphoma |
| Ellagic acid | Peripheral Nervous System Diseases |
| Ellagic acid | Smooth muscle hyperplasia |
| Ellagic acid | Macular Degeneration |
| Ellagic acid | Brain Cancer |
| Ellagic acid | Hepatocellular carcinoma |
| Ellagic acid | Hormone-refractory Prostate cancer |
| Ellagic acid | Kaposi's Sarcoma |
| Ellagic acid | Lung Cancer |
| Ellagic acid | Pancreatic Cancer |
| Ellagic acid | Renal Cell Carcinoma |
| Ellagic acid | Carcinoma, Non-Small-Cell Lung |
| Ellagic acid | Gastrointestinal Neoplasms |
| Ellagic acid | Head and Neck Neoplasms |
| Ellagic acid | Neoplasms |
| Ellagic acid | Neurotoxicity Syndromes |
| Ellagic acid | Neutropenia |
| Ellagic acid | Ovarian Neoplasms |
| Ellagic acid | Rectal Neoplasms |
| Ellagic acid | Anemia |
| Ellagic acid | Osteoarthritis |
| Ellagic acid | Breast Neoplasms |
| Ellagic acid | Drug Toxicity |
| Ellagic acid | Colorectal Neoplasms |
| Ellagic acid | Ovarian cancer |
| Ellagic acid | Chronic Myelogenous Leukemia (CML) |
| Ellagic acid | Hypospadias 1, X-linked |
| Ellagic acid | Melanoma |
| Ellagic acid | HER2-positive Metastatic Breast Cancer |
| Ellagic acid | Gastrointestinal Stromal Tumors (GIST) |
| Ellagic acid | Androgen insensitivity |
| Ellagic acid | Refractory Hematological Malignancies |
| Ellagic acid | Multiple Myeloma |
| Ellagic acid | Hematological Malignancies |
| Ellagic acid | B-cell malignancies |
| Ellagic acid | Cancer, unspecific |
| Ellagic acid | Postmenopausal symptoms |
| Ellagic acid | Viral infection, unspecified |
| Ellagic acid | Brain injury |
| Ellagic acid | Breast cancer |
| Ellagic acid | Coronary atherosclerosis |
| Ellagic acid | Neurodegenerative diseases |
| Ellagic acid | Osteoporosis, unspecified |
| Ellagic acid | Endocrine independent cancer |
| Ellagic acid | Solid tumors |
| Ellagic acid | Non-small Cell Lung Cancer |
| Ellagic acid | Non-Hodgkin's Lymphoma |
| Ellagic acid | Nasopharyngeal Cancer (NPC) |
| Ellagic acid | Hepatocellular Carcinoma (HCC) |
| Ellagic acid | Chronic lymphocytic leukemia (CLL) |
| Ellagic acid | Cardiovascular disease, unspecified |
| Ellagic acid | Advanced solid tumors |
| Ellagic acid | Acute myeloid leukemia (AML) |
| Ellagic acid | Acute lymphoblastic leukemia (ALL) |
| Ellagic acid | Migraine |
| Ellagic acid | Myocardial Infarction |
| Ellagic acid | Cardiovascular disease |
| Ellagic acid | Osteoporosis |
| Ellagic acid | XY disorders of sex development (Disorders in androgen synthesis or action) |
| Ellagic acid | Spinal and bulbar muscular atrophy |
| Ellagic acid | Spinal and bulbar muscular atrophy of Kennedy |
| Ellagic acid | Prostate cancer |
| Ellagic acid | XY disorders of sex development (Other) |
| Juglone | Bladder cancer |
| Juglone | Cancer, unspecific |
| Juglone | Hepatocellular carcinoma |
| Juglone | Kidney Cancer |
| Juglone | Prostate cancer |
| Juglone | Endometrial carcinoma |
| Juglone | Leukemia, Unspecified |
| Juglone | Malaria |
| Juglone | Mesothelioma |
| Juglone | Metastatic osteosarcoma in the lung |
| Juglone | Obesity |
| Juglone | Tumors |
| Juglone | Ischemic injury of the liver |
| Juglone | Heart Failure |
| Juglone | Precursor Cell Lymphoblastic Leukemia-Lymphoma |
| Juglone | Anemia due to disorders of glutathione metabolism |
| Juglone | Other phagocyte defects |
| Juglone | Microbial infections |
| Juglone | Allergic inflammation |
| Juglone | Asthma |
| Juglone | Asthmatic bronchial mucosal inflammation |
| Juglone | Parasitic diseases |
| Kaempferol | Spinal and bulbar muscular atrophy |
| Kaempferol | XY disorders of sex development (Other) |
| Kaempferol | lschemia reperfusion injuries |
| Kaempferol | Prostate cancer |
| Kaempferol | Cardiovascular disease, unspecifed |
| Kaempferol | Chronic inflammatory diseases |
| Kaempferol | XY disorders of sex development (Disorders in androgen synthesis or action) |
| Kaempferol | Spinal and bulbar muscular atrophy of Kennedy |
| Kaempferol | Hypospadias 1, X-linked |
| Kaempferol | Androgen insensitivity |
| Kaempferol | Adrenocorticotrophic hormone-secreting pituitary tumors |
| Kaempferol | Asthma |
| Kaempferol | Atherosclerosis |
| Kaempferol | Atopic Dermatitis |
| Kaempferol | Autoimmune Diseases |
| Kaempferol | Bladder cancer |
| Kaempferol | Crohns's Disease, unspecifled |
| Kaempferol | Diabetes mllitus |
| Kaempferol | Inflammation |
| Kaempferol | Inflammatory Bowel Disease |
| Kaempferol | Insulin resistance |
| Kaempferol | Ischemic heart disease |
| Kaempferol | Multiple Sclerosis |
| Kaempferol | Obesity |
| Kaempferol | Pancreatic Cancer |
| Kaempferol | Psoriasis |
| Kaempferol | Renal Cell Carcinoma |
| Kaempferol | Testicular cancer |
| Kaempferol | Thyroid follicular carcinoma |
| Kaempferol | Ulcerative colitis |
| Kaempferol | Abdominal aortic aneurysm |
| Kaempferol | Adenomatous polyposis |
| Kaempferol | Alzheimer's Disease |
| Kaempferol | Analgesics |
| Kaempferol | Arthritis |
| Kaempferol | Breast cancer |
| Kaempferol | Cancer, unspecific |
| Kaempferol | Carcinoma in situ, unspecified |
| Kaempferol | Carpal tunnel syndrome |
| Kaempferol | Colorectal cancer |
| Kaempferol | Dysmenorrhea, unspecified |
| Kaempferol | Endometriosis |
| Kaempferol | Genitourinary tumors |
| Kaempferol | Gestational hypertension |
| Kaempferol | Inflammatory diseases |
| Kaempferol | Lung Cancer |
| Kaempferol | Malignant mesothelioma |
| Kaempferol | Meningioma |
| Kaempferol | Myocardial Infarction |
| Kaempferol | Oropharyngeal squamous cell carcinoma |
| Kaempferol | Osteoarthritis |
| Kaempferol | Pain, unspecified |
| Kaempferol | Pathological angiogenesis |
| Kaempferol | Peutz-Jeghers syndrome |
| Kaempferol | Pyresis |
| Kaempferol | Rheumatoid arthritis, unspecified |
| Kaempferol | Stroke |
| Kaempferol | Vascular lesion regression |
| Kaempferol | Chronic Myelogenous Leukemia (CML) |
| Kaempferol | Gastrointestinal Stromal Tumors (GIST) |
| Kaempferol | Hematological Malignancies |
| Kaempferol | HER2-positive Metastatic Breast Cancer |
| Kaempferol | Melanoma |
| Kaempferol | Multiple Myeloma |
| Kaempferol | Non-small Cell Lung Cancer |
| Kaempferol | Ovarian cancer |
| Kaempferol | Refractory Hematological Malignancies |
| Kaempferol | Solid tumors |
| Kaempferol | Angioedema |
| Kaempferol | Heart Failure |
| Kaempferol | Not Available |
| Kaempferol | Malignancies |
| Kaempferol | Noninsulin-dependent diabetes mellitus |
| Kaempferol | Coagulative disorders |
| Kaempferol | Coronary atherosclerosis |
| Kaempferol | Gliomas |
| Kaempferol | Heparin-induced thrombocytopenia type II |
| Kaempferol | Multiple organ failure |
| Kaempferol | Thromboembolic disorders |
| Kaempferol | Thrombosis |
| Kaempferol | Thrombotic disease |
| Kaempferol | Bronchospasm (histamine induced) |
| Kaempferol | Cognitive deficits |
| Kaempferol | Schizophrenia |
| Kaempferol | Angina |
| Kaempferol | Colon cancer |
| Kaempferol | Coronary Artery Disease |
| Kaempferol | Helminth infection |
| Kaempferol | Hypertension, Angina |
| Kaempferol | Sepsis |
| Kaempferol | Anxiety disorder, unspecified |
| Kaempferol | Disorders of initiating and maintaining sleep [insomnias] |
| Kaempferol | Hypoxic-ischemic encephalopathy |
| Kaempferol | Motor neurone disease |
| Kaempferol | Parkinson's disease |
| Kaempferol | Blood group, Yt system |
| Kaempferol | Parkinson Disease |
| Kaempferol | Depression |
| Kaempferol | Autoimmune cardiomyopathy |
| Kaempferol | Bronchoconstriction (cold air-induced) |
| Kaempferol | Chronic obstructive pulmonary disease, unspecifled |
| Kaempferol | Hypothermia |
| Kaempferol | Neurogenic bladder |
| Kaempferol | Tremor, unspecified |
| Kaempferol | Shy-Drager syndrome |
| Kaempferol | Anxiety Disorders |
| Kaempferol | Insomnia |
| Kaempferol | Acute promyelocytic leukemia |
| Kaempferol | Bacterial Infections |
| Kaempferol | Fungal diseases |
| Kaempferol | Herpes virus infection |
| Kaempferol | L eishmania Infections |
| Kaempferol | Malaria |
| Kaempferol | Trichomoniasis |
| Kaempferol | Chronic lymphocytic leukemia |
| Kaempferol | Prostate cancer (hormone refractory) |
| Kaempferol | Waldenstrom's macroglobulinemia |
| Kaempferol | Behcet's disease |
| Kaempferol | Congestive Heart Failure |
| Kaempferol | Hyperimmunoglobulinemia D |
| Kaempferol | Periodic fever syndrome |
| Kaempferol | Rheumatic diseases |
| Kaempferol | Solid Tumor |
| Kaempferol | Allergic airway inflammation |
| Kaempferol | Cancer (multidrug resistant) |
| Kaempferol | Rheumatoid arthritis |
| Kaempferol | Vascular disease |
| Kaempferol | Hearing Loss |
| Kaempferol | Inflammatory Disorders, Unspecified |
| Kaempferol | Gout |
| Kaempferol | Chondrosarcoma |
| Kaempferol | Emphysema |
| Kaempferol | Hormone-refractory Prostate cancer |
| Kaempferol | Kaposi's Sarcoma |
| Kaempferol | Myocardial infarction (MI) |
| Kaempferol | Hypothalamic-pituitary ACTH function |
| Kaempferol | Abortion, Spontaneous |
| Kaempferol | Arthritis, Rheumatoid |
| Kaempferol | Breast Neoplasms |
| Kaempferol | Colorectal Neoplasms |
| Kaempferol | Fatigue |
| Kaempferol | Neural Tube Defects |
| Kaempferol | Ovarian Neoplasms |
| Kaempferol | Pulmonary Disease, Chronic Obstructive |
| Kaempferol | Tardive dyskinesia |
| Kaempferol | Tobacco Use Disorder |
| Kaempferol | Urinary Bladder Neoplasms |
| Kaempferol | Chronic Obstructive Pulmonary Disease (COPD) |
| Kaempferol | Inflammatory skin disorder |
| Kaempferol | Ischemic Stroke |
| Kaempferol | Psoriasis and Psoriatic Disorders |
| Kaempferol | Endotoxin-induced myocardial neutrophil accumulation and contractile dysfunction |
| Kaempferol | Adult respiratory distress syndrome |
| Kaempferol | Allergic diseases |
| Kaempferol | Allergic rhinitis, unspecified |
| Kaempferol | Antigen-induced decrease in coronary flow and cardiac anaphylaxis |
| Kaempferol | Bronchiolar carcinoma |
| Kaempferol | Cerebral vasospasm |
| Kaempferol | Cervical cancer |
| Kaempferol | Chronic myeloid leukemia |
| Kaempferol | Dermatological disorders |
| Kaempferol | Gastrointestinal Cancers |
| Kaempferol | Glomerulonephritis |
| Kaempferol | Inflammatory lung disease |
| Kaempferol | Magnesium deficiency dermatitis |
| Kaempferol | Nephrosis |
| Kaempferol | Polyarthritis, unspecified |
| Kaempferol | Prion diseases |
| Kaempferol | Pulmonary fibrosis |
| Kaempferol | Sjogren-Larsson syndrome |
| Kaempferol | Urological cancers |
| Kaempferol | Vasospasm |
| Kaempferol | Carcinoma, Non-Small-Cell Lung |
| Kaempferol | Drug Toxicity |
| Kaempferol | Gastrointestinal Neoplasms |
| Kaempferol | Head and Neck Neoplasms |
| Kaempferol | Neoplasms |
| Kaempferol | Neurotoxicity Syndromes |
| Kaempferol | Neutropenia |
| Kaempferol | Peripheral Nervous System Diseases |
| Kaempferol | Precursor Cell Lymphoblastic Leukemia-Lymphoma |
| Kaempferol | Rectal Neoplasms |
| Kaempferol | Head and Neck Cancer |
| Luteolin | Prostate cancer (metastatic) |
| Luteolin | Gastric Cancer |
| Luteolin | Rectal Neoplasms |
| Luteolin | Small cell lung cancer |
| Luteolin | Adenomatous polyposis |
| Luteolin | Stroke |
| Luteolin | Cardiovascular disease, unspecified |
| Luteolin | Malignancies |
| Luteolin | Osteoarthritis |
| Luteolin | Noninsulin-dependent diabetes mellitus |
| Luteolin | Bladder cancer |
| Luteolin | Carcinoma, Non-Small-Cell Lung |
| Luteolin | Dementia |
| Luteolin | Vascular lesion regression |
| Luteolin | Abdominal aortic aneurysm |
| Luteolin | Androgen insensitivity |
| Luteolin | Hepatocellular carcinoma |
| Luteolin | Pancreatic Cancer |
| Luteolin | Refractory Hematological Malignancies |
| Luteolin | Cancer, unspecific |
| Luteolin | Chronic inflammatory diseases |
| Luteolin | Solid tumors |
| Luteolin | Pyresis |
| Luteolin | Heart Failure |
| Luteolin | Periodic fever syndrome |
| Luteolin | Lung Cancer |
| Luteolin | Macular Degeneration |
| Luteolin | Precursor Cell Lymphoblastic Leukemia-Lymphoma |
| Luteolin | Peripheral Nervous System Diseases |
| Luteolin | Pain, unspecified |
| Luteolin | Ovarian cancer |
| Luteolin | Rheumatoid arthritis, unspecified |
| Luteolin | Analgesics |
| Luteolin | Insulin-dependent diabetes mellitus |
| Luteolin | Malignant mesothelioma |
| Luteolin | Meningioma |
| Luteolin | Proliferative diseases |
| Luteolin | Angioedema |
| Luteolin | XY disorders of sex development (Disorders in androgen synthesis or action) |
| Luteolin | Prostate cancer |
| Luteolin | Spinal and bulbar muscular atrophy |
| Luteolin | XY disorders of sex development (Other) |
| Luteolin | Spinal and bulbar muscular atrophy of Kennedy |
| Luteolin | Hypospadias 1, X-linked |
| Luteolin | Endometriosis |
| Luteolin | Renal Cell Carcinoma |
| Luteolin | Alzheimer's Disease |
| Luteolin | Chondrosarcoma |
| Luteolin | Arthritis |
| Luteolin | Breast cancer |
| Luteolin | Carcinoma in situ, unspecified |
| Luteolin | Carpal tunnel syndrome |
| Luteolin | Colorectal cancer |
| Luteolin | Dysmenorrhea, unspecified |
| Luteolin | Gestational hypertension |
| Luteolin | Inflammation |
| Luteolin | Myocardial Infarction |
| Luteolin | Oropharyngeal squamous cell carcinoma |
| Luteolin | Pathological angiogenesis |
| Luteolin | Peutz-Jeghers syndrome |
| Luteolin | Kaposi's Sarcoma |
| Luteolin | Chronic Myelogenous Leukemia (CML) |
| Luteolin | Gastrointestinal Stromal Tumors (GIST) |
| Luteolin | Hematological Malignancies |
| Luteolin | HER2-positive Metastatic Breast Cancer |
| Luteolin | Melanoma |
| Luteolin | Multiple Myeloma |
| Luteolin | Non-small Cell Lung Cancer |
| Luteolin | Glioblastoma multiforme |
| Luteolin | Not Available |
| Luteolin | Autoimmune Diseases |
| Luteolin | Diabetes mellitus |
| Luteolin | Obesity |
| Luteolin | HCV infection |
| Luteolin | Head and neck tumors |
| Luteolin | Lymphangiomatosis |
| Luteolin | Solid Tumor |
| Luteolin | Squamous cell carcinoma |
| Luteolin | Tumors |
| Luteolin | Colorectal Neoplasms |
| Luteolin | Brain Cancer |
| Luteolin | Hormone-refractory Prostate cancer |
| Luteolin | Smooth muscle hyperplasia |
| Luteolin | Neurodegenerative diseases |
| Luteolin | Asthma |
| Luteolin | Behcet's disease |
| Luteolin | Congestive Heart Failure |
| Luteolin | Crohns's Disease, unspecifled |
| Luteolin | Hyperimmunoglobulinemia D |
| Luteolin | Rheumatic diseases |
| Luteolin | Allergic airway inflammation |
| Luteolin | Cancer (multidrug resistant) |
| Luteolin | Rheumatoid arthritis |
| Luteolin | Vascular disease |
| Luteolin | Cachexia |
| Luteolin | Hyperinflammatory provoked organ injury |
| Luteolin | Inflammatory Disorders, Unspecified |
| Luteolin | Osteoporosis, unspecified |
| Luteolin | Systemic-onset juvenile idiopathic arthritis |
| Luteolin | Kidney Cancer |
| Luteolin | Gout |
| Luteolin | Emphysema |
| Luteolin | Myocardial infarction (MI) |
| Luteolin | Multiple Sclerosis |
| Luteolin | Psoriasis and Psoriatic Disorders |
| Luteolin | Breast Neoplasms |
| Luteolin | Inflammatory diseases |
| Luteolin | Drug Toxicity |
| Luteolin | Gastrointestinal Neoplasms |
| Luteolin | Head and Neck Neoplasms |
| Luteolin | Neoplasms |
| Luteolin | Neurotoxicity Syndromes |
| Luteolin | Neutropenia |
| Luteolin | Ovarian Neoplasms |
| Luteolin | Genitourinary tumors |
| Quercetin | Cardiovascular disease, unspecifed |
| Quercetin | Chronic inflammatory diseases |
| Quercetin | Prostate cancer |
| Quercetin | Spinal and bulbar muscular atrophy |
| Quercetin | XY disorders of sex development (Other) |
| Quercetin | XY disorders of sex development (Disorders in androgen synthesis or action) |
| Quercetin | Spinal and bulbar muscular atrophy of Kennedy |
| Quercetin | Hypospadias 1, X-linked |
| Quercetin | Androgen insensitivity |
| Quercetin | Adrenocorticotrophic hormone-secreting pituitary tumors |
| Quercetin | Asthma |
| Quercetin | Atherosclerosis |
| Quercetin | Atopic Dermatitis |
| Quercetin | Autoimmune Diseases |
| Quercetin | Bladder cancer |
| Quercetin | Crohns's Disease, unspecified |
| Quercetin | Diabetes mellitus |
| Quercetin | Inflammation |
| Quercetin | Inflammatory Bowel Disease |
| Quercetin | Insulin resistance |
| Quercetin | Ischemic heart disease |
| Quercetin | Multiple Sclerosis |
| Quercetin | Obesity |
| Quercetin | Pancreatic Cancer |
| Quercetin | Psoriasis |
| Quercetin | Renal Cell Carcinoma |
| Quercetin | Testicular cancer |
| Quercetin | Thyroid follicular carcinoma |
| Quercetin | Ulcerative colitis |
| Quercetin | Abdominal aortic aneurysm |
| Quercetin | Adenomatous polyposis |
| Quercetin | Alzheimer's Disease |
| Quercetin | Analgesics |
| Quercetin | Arthritis |
| Quercetin | Breast cancer |
| Quercetin | Cancer, unspecific |
| Quercetin | Carcinoma in situ, unspecified |
| Quercetin | Carpal tunnel syndrome |
| Quercetin | Colorectal cancer |
| Quercetin | Dysmenorrhea, unspecified |
| Quercetin | Endometriosis |
| Quercetin | Genitourinary tumors |
| Quercetin | Gestational hypertension |
| Quercetin | Inflammatory diseases |
| Quercetin | Lung Cancer |
| Quercetin | Malignant mesothelioma |
| Quercetin | Meningioma |
| Quercetin | Myocardial Infarction |
| Quercetin | Oropharyngeal squamous cell carcinoma |
| Quercetin | Osteoarthritis |
| Quercetin | Pain, unspecified |
| Quercetin | Pathological angiogenesis |
| Quercetin | Peutz-Jeghers syndrome |
| Quercetin | Pyresis |
| Quercetin | Rheumatoid arthritis, unspecifed |
| Quercetin | Stroke |
| Quercetin | Vascular lesion regression |
| Quercetin | Chronic Myelogenous Leukemia (CML) |
| Quercetin | Gastrointestinal Stromal Tumors (GIST) |
| Quercetin | Hematological Malignancies |
| Quercetin | HER2-positive Metastatic Breast Cancer |
| Quercetin | Melanoma |
| Quercetin | Multiple Myeloma |
| Quercetin | Non-small Cell Lung Cancer |
| Quercetin | Ovarian cancer |
| Quercetin | Refractory Hematological Malignancies |
| Quercetin | Solid tumors |
| Quercetin | Angioedema |
| Quercetin | Heart Failure |
| Quercetin | Malignancies |
| Quercetin | Noninsulin-dependent diabetes melitus |
| Quercetin | Diabetic complications |
| Quercetin | Diabetic neuropathy |
| Quercetin | Diabetic retinopathy |
| Quercetin | Neuropathic pain |
| Quercetin | Acute promyelocytic leukemia |
| Quercetin | Bacterial Infections |
| Quercetin | Fungal diseases |
| Quercetin | Herpes virus infection |
| Quercetin | Leishmania Infections |
| Quercetin | Malaria |
| Quercetin | Trichomoniasis |
| Quercetin | Coagulative disorders |
| Quercetin | Coronary atherosclerosis |
| Quercetin | Gliomas |
| Quercetin | Heparin-induced thrombocytopenia type II |
| Quercetin | Multiple organ failure |
| Quercetin | Thromboembolic disorders |
| Quercetin | Thrombosis |
| Quercetin | Thrombotic disease |
| Quercetin | Cardiac arrhythmias |
| Quercetin | Cardiac dysrhythmias |
| Quercetin | Epileptic seizures |
| Quercetin | Pain |
| Quercetin | Refractory partial epilepsy |
| Quercetin | Sustained ventricular tachycardia |
| Quercetin | Atrial fibrillation and flutter |
| Quercetin | Thromboembolism |
| Quercetin | Anxiety disorder, unspecified |
| Quercetin | Chronic obstructive pulmonary disease, unspecified |
| Quercetin | Depression |
| Quercetin | Glaucoma |
| Quercetin | Hypertension |
| Quercetin | Obstructive airway disease |
| Quercetin | Respiratory distress syndrome |
| Quercetin | Skeletal muscle wasting |
| Quercetin | Skeletal muscle weakness |
| Quercetin | Brain Cancer |
| Quercetin | Myocardial infarction (MI) |
| Quercetin | Not Available |
| Quercetin | Angina |
| Quercetin | Colon cancer |
| Quercetin | Coronary Artery Disease |
| Quercetin | Helminth infection |
| Quercetin | Hypertension, Angina |
| Quercetin | Schizophrenia |
| Quercetin | Sepsis |
| Quercetin | Cognitive deficits |
| Quercetin | Hypoxic-ischemic encephalopathy |
| Quercetin | Motor neurone disease |
| Quercetin | Parkinson's disease |
| Quercetin | Blood group, Yt system |
| Quercetin | Parkinson Disease |
| Quercetin | Anxiety Disorders |
| Quercetin | Insomnia |
| Quercetin | Major Depressive Disorder |
| Quercetin | Neurological diseases |
| Quercetin | Glioblastoma multiforme |
| Quercetin | HCV infection |
| Quercetin | Head and neck tumors |
| Quercetin | Lymphangiomatosis |
| Quercetin | Solid Tumor |
| Quercetin | Squamous cell carcinoma |
| Quercetin | Tumors |
| Quercetin | Colorectal Neoplasms |
| Quercetin | Macular Degeneration |
| Quercetin | Chronic lymphocytic leukemia |
| Quercetin | Prostate cancer (hormone refractory) |
| Quercetin | Waldenstrom's macroglobulinemia |
| Quercetin | Hepatocellular carcinoma |
| Quercetin | Hormone-refractory Prostate cancer |
| Quercetin | Kaposi's Sarcoma |
| Quercetin | Smooth muscle hyperplasia |
| Quercetin | Neurodegenerative diseases |
| Quercetin | Proliferative diseases |
| Quercetin | Rectal Neoplasms |
| Quercetin | Behcet's disease |
| Quercetin | Congestive Heart Failure |
| Quercetin | Hyperimmunoglobulinemia D |
| Quercetin | Peridic fever syndrome |
| Quercetin | Rheumatic diseases |
| Quercetin | Allergic airway inflammation |
| Quercetin | Cancer (multidrug resistant) |
| Quercetin | Rheumatoid arthritis |
| Quercetin | Vascular disease |
| Quercetin | Cachexia |
| Quercetin | Hyperinflammatory provoked organ injury |
| Quercetin | Inflammatory Disorders, Unspecified |
| Quercetin | Osteoporosis, unspecified |
| Quercetin | Systemic-onset juvenile idiopathic arthritis |
| Quercetin | Kidney Transplantation |
| Quercetin | African trypanosomiasis |
| Quercetin | Hereditary Polyposis Syndromes |
| Quercetin | Sleeping Sickness |
| Quercetin | Gout |
| Quercetin | lschemic injury of the liver |
| Quercetin | Chondrosarcoma |
| Quercetin | Emphysema |
| Quercetin | Hypothalamic-pituitary ACTH function |
| Quercetin | Abortion, Spontaneous |
| Quercetin | Arthritis, Rheumatoid |
| Quercetin | Breast Neoplasms |
| Quercetin | Fatigue |
| Quercetin | Neural Tube Defects |
| Quercetin | Ovarian Neoplasms |
| Quercetin | Pulmonary Disease, Chronic Obstructive |
| Quercetin | Tardive dyskinesia |
| Quercetin | Tobacco Use Disorder |
| Quercetin | Urinary Bladder Neoplasms |
| Quercetin | Coronary syndromes |
| Quercetin | Disseminated intravascular coagulation |
| Quercetin | Over-expression of TF |
| Quercetin | Heart disease, unspecified |
| Quercetin | Acne |
| Quercetin | Chronic hepatitis C |
| Quercetin | Pediatric |
| Quercetin | Psoriasis and Psoriatic Disorders |
| Quercetin | Acute coronary syndromes |
| Quercetin | Inflammatory diseases associated with hypoxia |
| Quercetin | Peyronie's disease |
| Quercetin | Renal ischemia-reperfusion injury |
| Quercetin | Chronic Obstructive Pulmonary Disease (COPD) |
| Quercetin | Inflammatory skin disorder |
| Quercetin | Ischemic Stroke |
| Quercetin | Endotoxin-induced myocardial neutrophil accumulation and contractile dysfunction |
| Quercetin | Peripheral Vascular Disease |
| Quercetin | Endometrial Neoplasms |
| Quercetin | Radiation enteropathy |
| Quercetin | Bone Diseases, Metabolic |
| Quercetin | Fractures, Bone |
| Quercetin | Osteonecrosis |
| Quercetin | Osteoporosis |
| Quercetin | Adult respiratory distress syndrome |
| Quercetin | Allergic diseases |
| Quercetin | Allergic rhinitis, unspecified |
| Quercetin | Antigen-induced decrease in coronary flow and cardiac anaphylaxis |
| Quercetin | Bronchiolar carcinoma |
| Quercetin | Cerebral vasospasm |
| Quercetin | Cervical cancer |
| Quercetin | Chronic myeloid leukemia |
| Quercetin | Dermatological disorders |
| Quercetin | Gastrointestinal Cancers |
| Quercetin | Glomerulonephritis |
| Quercetin | Inflammatory lung disease |
| Quercetin | Magnesium deficiency dermatitis |
| Quercetin | Nephrosis |
| Quercetin | Polyarthritis, unspecified |
| Quercetin | Prion diseases |
| Quercetin | Pulmonary flbrosis |
| Quercetin | Sjogren-Larsson syndrome |
| Quercetin | Urological cancers |
| Quercetin | Vasospasm |
| Quercetin | Carcinoma, Non-Small-Cell Lung |
| Quercetin | Drug Toxicity |
| Quercetin | Gastrointestinal Neoplasms |
| Quercetin | Head and Neck Neoplasms |
| Quercetin | Neoplasms |
| Quercetin | Neurotoxicity Syndromes |
| Quercetin | Neutropenia |
| Quercetin | Peripheral Nervous System Diseases |
| Quercetin | Precursor Cell Lymphoblastic L eukemia-Lymphoma |
| Sinapic acid | Vascular lesion regression |
| Sinapic acid | Cardiovascular disease, unspecified |
| Sinapic acid | Carpal tunnel syndrome |
| Sinapic acid | Colorectal cancer |
| Sinapic acid | Multiple Sclerosis |
| Sinapic acid | lschemia reperfusion injuries |
| Sinapic acid | Abdominal aortic aneurysm |
| Sinapic acid | Analgesics |
| Sinapic acid | Bladder cancer |
| Sinapic acid | Carcinoma in situ, unspecified |
| Sinapic acid | Respiratory distress syndrome |
| Sinapic acid | Gestational hypertension |
| Sinapic acid | Inflammation |
| Sinapic acid | Inflammatory diseases |
| Sinapic acid | Lung Cancer |
| Sinapic acid | Meningioma |
| Sinapic acid | Oropharyngeal squamous cell carcinoma |
| Sinapic acid | Breast cancer |
| Sinapic acid | Pain, unspecified |
| Sinapic acid | Peutz-Jeghers syndrome |
| Sinapic acid | Pyresis |
| Sinapic acid | Renal Cell Carcinoma |
| Sinapic acid | Angina |
| Sinapic acid | Prostate cancer |
| Sinapic acid | Hypertension, Angina |
| Sinapic acid | Schizophrenia |
| Sinapic acid | Sepsis |
| Sinapic acid | Anxiety disorder, unspecified |
| Sinapic acid | Asthma |
| Sinapic acid | Cardiac arrhythmias |
| Sinapic acid | Chronic obstructive pulmonary disease, unspecified |
| Sinapic acid | Depression |
| Sinapic acid | Glaucoma |
| Sinapic acid | Hypertension |
| Sinapic acid | Skeletal muscle wasting |
| Sinapic acid | Skeletal muscle weakness |
| Sinapic acid | Insomnia |
| Sinapic acid | Major Depressive Disorder |
| Sinapic acid | Neurological diseases |
| Sinapic acid | Parkinson's disease |
| Sinapic acid | Arthritis |
| Sinapic acid | Stroke |
| Sinapic acid | Endometriosis |
| Sinapic acid | Rheumatoid arthritis, unspecified |
| Sinapic acid | Pathological angiogenesis |
| Sinapic acid | Adenomatous polyposis |
| Sinapic acid | Alzheimer's Disease |
| Sinapic acid | Myocardial Infarction |
| Sinapic acid | Helminth infection |
| Sinapic acid | Dysmenorrhea, unspecified |
| Sinapic acid | Colon cancer |
| Sinapic acid | Osteoarthritis |
| Sinapic acid | Coronary Artery Disease |
| Sinapic acid | Malignant mesothelioma |
| Sinapic acid | Genitourinary tumors |
| Sinapic acid | Cancer, unspecific |
| Sinapic acid | Obstructive airway disease |
| Sinapic acid | Anxiety Disorders |
| Sinapic acid | Chronic inflammatory diseases |

**Table S4 Related targets of 11 active substances**

| Compound | Related targets | Abbreviation |
| --- | --- | --- |
| (-)-Epicatechin | Urokinase-type plasminogen activator | Upa |
| (-)-Epicatechin | Tumor necrosis factor | TNF |
| (-)-Epicatechin | Transcription factor AP-1 | AP-1 |
| (-)-Epicatechin | Tissue-type plasminogen activator | t-PA |
| (-)-Epicatechin | Retinoic acid receptor RXR-alpha | RXR-α |
| (-)-Epicatechin | RAC-alpha serine/threonine-protein kinase | RXC-α |
| (-)-Epicatechin | Prostaglandin G/H synthase 2 | PTGS2 |
| (-)-Epicatechin | Prostaglandin G/H synthase 1 | PTGS1 |
| (-)-Epicatechin | Nuclear receptor coactivator 2 | NCOA2 |
| (-)-Epicatechin | NADPH--cytochrome P450 reductase | NADPH |
| (-)-Epicatechin | mRNA of PKA Catalytic Subunit C-alpha | PKA C-α |
| (-)-Epicatechin | Interleukin-2 | IL-2 |
| (-)-Epicatechin | Heat shock protein HSP 90 | Hsp90 |
| (-)-Epicatechin | Glutathione synthetase | GS |
| (-)-Epicatechin | Glutamate--cysteine ligase catalytic subunit | GCLCE |
| (-)-Epicatechin | Glutamate receptor 2 | NR2B |
| (-)-Epicatechin | Glutamate [NMDA] receptor subunit zeta-1 | NMDA1 |
| (-)-Epicatechin | Estrogen receptor | ER |
| (-)-Epicatechin | C-C motif chemokine 2 | CCL2 |
| (-)-Epicatechin | Calmodulin | CaM |
| (-)-Epicatechin | Beta-lactamase | ESBLs |
| (-)-Epicatechin | Angiotensin-convering enzyme | ACE |
| (-)-Epigallocatechin gallate | Xanthine dehydrogenase/oxidase | XDH |
| (-)-Epigallocatechin gallate | Vascular endothelial growth factor receptor 2 | VEGFA2 |
| (-)-Epigallocatechin gallate | Vascular endothelial growth factor receptor 1 | VEGFA1 |
| (-)-Epigallocatechin gallate | Vascular endothelial growth factor A | VEGFA |
| (-)-Epigallocatechin gallate | Urokinase-type plasminogen activator | Upa |
| (-)-Epigallocatechin gallate | Tumor necrosis factor | TNF |
| (-)-Epigallocatechin gallate | Transcription factor AP-1 | AP-1 |
| (-)-Epigallocatechin gallate | Toll-like receptor 4 | TLR4 |
| (-)-Epigallocatechin gallate | T-cell surface glycoprotein CD4 | CD4 |
| (-)-Epigallocatechin gallate | Superoxide dismutase [Cu-Zn] | SOD |
| (-)-Epigallocatechin gallate | Stromelysin-1 | SL-1 |
| (-)-Epigallocatechin gallate | Serum amyloid A protein | SAA |
| (-)-Epigallocatechin gallate | Ribosomal protein S6 kinase alpha-1 | RPS6Kα1 |
| (-)-Epigallocatechin gallate | Retinoblastoma-associated protein | RP |
| (-)-Epigallocatechin gallate | Prostaglandin G/H synthase 2 | PTGS2 |
| (-)-Epigallocatechin gallate | Pro-epidermal growth factor | EGF |
| (-)-Epigallocatechin gallate | Ornithine decarboxylase | ODC |
| (-)-Epigallocatechin gallate | Nitric oxide synthase, inducible | iNOS |
| (-)-Epigallocatechin gallate | NADPH--cytochrome P450 reductase | NADPH |
| (-)-Epigallocatechin gallate | NADP-dependent malic enzyme | NADP-ME |
| (-)-Epigallocatechin gallate | Mitogen-activated protein kinase 8 | MAPK8 |
| (-)-Epigallocatechin gallate | Mitogen-activated protein kinase 3 | MAPK3 |
| (-)-Epigallocatechin gallate | Mitogen-activated protein kinase 1 | MAPK1 |
| (-)-Epigallocatechin gallate | Matrix metalloproteinase-14 | MMP14 |
| (-)-Epigallocatechin gallate | Matrilysin | MAT |
| (-)-Epigallocatechin gallate | Low-density lipoprotein receptor | LDL |
| (-)-Epigallocatechin gallate | Interstitial collagenase | CLG |
| (-)-Epigallocatechin gallate | Interleukin-6 | IL-6 |
| (-)-Epigallocatechin gallate | High affinity immunoglobulin epsilon receptor subunit alpha | FCER1A |
| (-)-Epigallocatechin gallate | Heparin-binding growth factor 2 | HBGF2 |
| (-)-Epigallocatechin gallate | Glucose-6-phosphate 1-dehydrogenase | G6PD |
| (-)-Epigallocatechin gallate | Fatty acid synthase | FAS |
| (-)-Epigallocatechin gallate | Estrogen receptor | ER |
| (-)-Epigallocatechin gallate | Epidermal growth factor receptor | EGFR |
| (-)-Epigallocatechin gallate | Ephrin type-A receptor 2 | EphA2 |
| (-)-Epigallocatechin gallate | Endothelin-1 receptor | ET-1 |
| (-)-Epigallocatechin gallate | Dual specificity mitogen-activated protein kinase kinase 1 | MAP2K1 |
| (-)-Epigallocatechin gallate | DNA topoisomerase 1 | TOPO 1 |
| (-)-Epigallocatechin gallate | Dihydrofolate reductase | DHFR |
| (-)-Epigallocatechin gallate | Collagenase 3 | TYPE 3 |
| (-)-Epigallocatechin gallate | Cellular tumor antigen p53 | TP53 |
| (-)-Epigallocatechin gallate | Cell division protein kinase 6 | CDK6 |
| (-)-Epigallocatechin gallate | Cell division protein kinase 4 | CDK4 |
| (-)-Epigallocatechin gallate | Cell division control protein 2 homolog | CDC2 |
| (-)-Epigallocatechin gallate | Catalase | CAT |
| (-)-Epigallocatechin gallate | Basic filbroblast growth factor receptor 1 | Bfgf |
| (-)-Epigallocatechin gallate | Apoptosis regulator Bcl-2 | BCL2 |
| (-)-Epigallocatechin gallate | Amyloid beta A4 protein | APB |
| (-)-Epigallocatechin gallate | AcetyI-CoA carboxylase 1 | ACC1 |
| (-)-Epigallocatechin gallate | 78 kDa glucose-regulated protein | GRP78 |
| (-)-Epigallocatechin gallate | 72 kDa type IV collagenase | TBE-1 |
| (-)-Epigallocatechin gallate | 3-oxo-5-alpha-steroid 4-dehydrogenase 2 | SRD5A2 |
| (+)-Catechin | Retinoic acid receptor RXR-alpha | RXR-α |
| (+)-Catechin | Prostaglandin G/H synthase 2 | PTGS2 |
| (+)-Catechin | Prostaglandin G/H synthase 1 | PTGS1 |
| (+)-Catechin | Nuclear receptor coactivator 2 | NCOA2 |
| (+)-Catechin | mRNA of PKA Catalytic Subunit C-alpha | PKA C-α |
| (+)-Catechin | Heat shock protein HSP 90 | Hsp90 |
| (+)-Catechin | Estrogen receptor | ER |
| (+)-Catechin | Catalase | CAT |
| (+)-Catechin | Calmodulin | CaM |
| (+)-Catechin | Beta-lactamase | ESBLs |
| Catechin gallate | Coagulation factor VI | FVI |
| Dihydroquercetin | Retinoic acid receptor RXR-alpha | RXR-α |
| Dihydroquercetin | Prostaglandin G/H synthase 2 | PTGS2 |
| Dihydroquercetin | Prostaglandin G/H synthase 1 | PTGS1 |
| Dihydroquercetin | Phosphatidylinositol-4,5-bisphosphate 3-kinase catalytic subunit, gamma isoform | PIK3CG |
| Dihydroquercetin | Heat shock protein HSP 90 . | Hsp90 |
| Dihydroquercetin | Aldose reductase | ARI |
| Ellagic acid | Vascular endothelial growth factor A | VEGFA |
| Ellagic acid | Progesterone receptor | PR |
| Ellagic acid | Heat shock protein HSP 90 | Hsp90 |
| Ellagic acid | Glutathione S-transferase P | GST P |
| Ellagic acid | Glutathione S-transferase Mu 2 | GST Mu 2 |
| Ellagic acid | Glutathione S-transferase Mu 1 | GST Mu 1 |
| Ellagic acid | Glutathione S-transferase A2 | GST A2 |
| Ellagic acid | Glutathione S-transferase A1 | GST A1 |
| Ellagic acid | Estrogen receptor | ER |
| Ellagic acid | Cell division protein kinase 2 | CDK2 |
| Ellagic acid | Androgen receptor | AR |
| Ellagic acid | 72 kDa type IV collagenase | TBE-1 |
| Juglone | Superoxide dismutase [Cu-Zn] | SOD |
| Juglone | Interleukin-5 | IL-5 |
| Juglone | Glutathione reductase, mitochondrial | GSR |
| Juglone | Glucose-6-phosphate 1-dehydrogenase | G6PD |
| Juglone | Fatty acid synthase | FAS |
| Juglone | Cystathionine gamma-synthase | CGS |
| Juglone | Cellular tumor antigen p53 | TP53 |
| Juglone | Catalase | CAT |
| Kaempferol | Xanthine dehydrogenase/oxidase | XDH |
| Kaempferol | Vascular cell adhesion protein 1 | VCAM-1 |
| Kaempferol | Tumor necrosis factor | TNF |
| Kaempferol | Trypsin-1 | TRY-1 |
| Kaempferol | Transcription factor AP-1 | AP-1 |
| Kaempferol | Thrombin | THr |
| Kaempferol | Sodium-dependent noradrenaline transporter | SLC6A2) |
| Kaempferol | Serine/threonine-protein phosphatase 2B catalytic subunit alpha isoform | CAM-PRP |
| Kaempferol | Prostaglandin G/H synthase 2 | PTGS2 |
| Kaempferol | Prostaglandin G/H synthase 1 | PTGS1 |
| Kaempferol | Progesterone receptor | PR |
| Kaempferol | Phosphatidylinositol-4,5-bisphosphate 3-kinase catalytic subunit, gamma isoform | PIK3CG |
| Kaempferol | Peroxisome proliferator activated receptor gamma | PPARg |
| Kaempferol | Nuclear receptor coactivator 2 | NCOA2 |
| Kaempferol | Nitric-oxide synthase, endothelial | eNOS |
| Kaempferol | Nitric oxide synthase, inducible | iNOS |
| Kaempferol | Muscarinic acetylcholine receptor M2 | mAChRM2 |
| Kaempferol | Muscarinic acetylcholine receptor M1 | mAChRM1 |
| Kaempferol | mRNA of PKA Catalytic Subunit C-alpha | PKA C-α |
| Kaempferol | Mitogen-activated protein kinase 8 | MAPK8 |
| Kaempferol | Interstitial collagenase | CLG |
| Kaempferol | Insulin receptor | IR |
| Kaempferol | Heme oxygenase 1 | HO-1 |
| Kaempferol | Heat shock protein HSP 90 | Hsp90 |
| Kaempferol | Glutathione S-transferase P | GST P |
| Kaempferol | Glutathione S-transferase Mu 2 | GST Mu 2 |
| Kaempferol | Glutathione S-transferase Mu 1 | GST Mu 1 |
| Kaempferol | Gamma-aminobutyric-acid receptor alpha-2 subunit | GABAα2 |
| Kaempferol | Gamma-aminobutyric acid receptor subunit alpha-1 | GABAα1 |
| Kaempferol | E-selectin | ELAM1 |
| Kaempferol | DNA topoisomerase II | TOPO II |
| Kaempferol | Dipeptidyl peptidase IV | DPP4 |
| Kaempferol | Cytochrome P450 3A4 | CYP3A4 |
| Kaempferol | Cytochrome P450 1A2 | CYP1A2 |
| Kaempferol | Coagulation factor VI | FVI |
| Kaempferol | Cell division control protein 2 homolog | CDC2 |
| Kaempferol | Calmodulin | CaM |
| Kaempferol | Aryl hydrocarbon receptor | AHR |
| Kaempferol | Arachidonate 5-lipoxygenase | 5-LO |
| Kaempferol | Apoptosis regulator BcI-2 | BCL2 |
| Kaempferol | Androgen receptor | AR |
| Kaempferol | Alpha-1B adrenergic receptor | Alpha-1B |
| Kaempferol | Aldo-keto reductase family 1 member C3 | AKR1C3 |
| Kaempferol | Acetylcholinesterase | AcCHS |
| Luteolin | Xanthine dehydrogenase/oxidase | XDH |
| Luteolin | Vascular endothelial growth factorA | VEGFA |
| Luteolin | Tumor necrosis factor | TNF |
| Luteolin | Trypsin-1 | TRY-1 |
| Luteolin | Transcription factor AP-1 | AP-1 |
| Luteolin | Retinoblastoma-associated protein | RP |
| Luteolin | Prostaglandin G/H synthase 2 | PTGS2 |
| Luteolin | Prostaglandin G/H synthase 1 | PTGS1 |
| Luteolin | Phosphatidylinositol-4,5-bisphosphate 3-kinase catalytic subunit, gamma isoform | PIK3CG |
| Luteolin | Nuclear receptor coactivator 2 | NCOA2 |
| Luteolin | mRNA of PKA Catalytic Subunit C-alpha | PKA C-α |
| Luteolin | Mitogen-activated protein kinase 1 | MAPK1 |
| Luteolin | Interstitial collagenase | CLG |
| Luteolin | Interleukin-6 | IL-6 |
| Luteolin | Interleukin-2 | IL-2 |
| Luteolin | Interferon gamma | IFN-γ |
| Luteolin | Insulin receptor | IR |
| Luteolin | Hepatocyte growth factor receptor | HGF |
| Luteolin | Heme oxygenase 1 | HO-1 |
| Luteolin | Heat shock protein HSP 90 | Hsp90 |
| Luteolin | Glutathione S-transferase P | GST P |
| Luteolin | Epidermal growth factor receptor | EGFR |
| Luteolin | DNA topoisomerase 1 | TOPO 1 |
| Luteolin | Dipeptidyl peptidase IV | DPP4 |
| Luteolin | Cellular tumor antigen p53 | TP53 |
| Luteolin | Cell division protein kinase 4 | CDK4 |
| Luteolin | Caspase-7 | CASP7 |
| Luteolin | Androgen receptor | AR |
| Luteolin | Amyloid beta A4 protein | APB |
| Luteolin | 72 kDa type IV collagenase | TBE-1 |
| Quercetin | Xanthine dehydrogenase/oxidase | XDH |
| Quercetin | Vascular endothelial growth factor A | VEGFA |
| Quercetin | Vascular cell adhesion protein 1 | VCAM-1 |
| Quercetin | Urokinase-type plasminogen activator | Upa |
| Quercetin | Tumor necrosis factor | TNF |
| Quercetin | Trypsin-1 | TRY-1 |
| Quercetin | Transcription factor AP-1 | AP-1 |
| Quercetin | Tissue-type plasminogen activator | t-PA |
| Quercetin | Tissue factor | TF |
| Quercetin | Thrombomodulin | TM |
| Quercetin | Thrombin | THr |
| Quercetin | Superoxide dismutase [Cu-Zn] | SOD |
| Quercetin | Stromelysin-1 | SL-1 |
| Quercetin | Sodium channel protein type 5 subunit alpha | SCN5A |
| Quercetin | Serum paraoxonase/arylesterase 1 | PON1 |
| Quercetin | Retinoic acid receptor RXR-alpha | RXR-α |
| Quercetin | Retinoblastoma-associated protein | RP |
| Quercetin | Prostatic acid phosphatase | PAP |
| Quercetin | Prostaglandin G/H synthase 2 | PTGS2 |
| Quercetin | Prostaglandin G/H synthase 1 | PTGS1 |
| Quercetin | Prostaglandin E2 receptor EP3 subtype | PTGER3 |
| Quercetin | Pro-epidermal growth factor | EGF |
| Quercetin | Potassium voltage-gated channel subfamily H member 2 | KCNS2 |
| Quercetin | Phosphatidylinositol-4. 5-bisphosphate 3-kinase catalytic subunit, gamma isoform | PIK3CG |
| Quercetin | Peroxisome proliferator activated receptor gamma | PPARg |
| Quercetin | Ornithine decarboxylase | ODC |
| Quercetin | Nuclear receptor coactivator 2 | NCOA2 |
| Quercetin | Nitric-oxide synthase, endothelial | eNOS |
| Quercetin | NADPH--cytochrome P450 reductase | NADPH |
| Quercetin | NAD(P)H dehydrogenase [quinone] 1 | NQO1 |
| Quercetin | Myeloperoxidase | MPO |
| Quercetin | mRNA of PKA Catalytic Subunit C-alpha | PKA C-α |
| Quercetin | Mitogen-activated protein kinase 1 | MAPK1 |
| Quercetin | Maltase-glucoamylase, intestinal | MGA |
| Quercetin | Interstitial collagenase | CLG |
| Quercetin | Interleukin-6 | IL-6 |
| Quercetin | Interleukin-2 | IL-2 |
| Quercetin | Interleukin-1 beta | IL-1β |
| Quercetin | Interferon gamma | IFN-γ |
| Quercetin | Insulin receptor | IR |
| Quercetin | Heme oxygenase 1 | HO-1 |
| Quercetin | Heat shock protein HSP 90 | Hsp90 |
| Quercetin | Glutathione S-transferase P | GST P |
| Quercetin | Glutathione S-transferase Mu 2 | GST Mu 2 |
| Quercetin | Glutathione S-transferase Mu 1 | GST Mu 1 |
| Quercetin | Gap junction alpha-1 protein | GJA1 |
| Quercetin | Gamma-aminobutyric acid receptor subunit alpha-1 | GABAα1 |
| Quercetin | Estrogen sulfotransferase | EST |
| Quercetin | E-selectin | ELAM1 |
| Quercetin | Epidermal growth factor receptor | EGFR |
| Quercetin | DNA topoisomerase II | TOPO II |
| Quercetin | DNA topoisomerase 1 | TOPO 1 |
| Quercetin | DNA gyrase subunit B | gyrB |
| Quercetin | DipeptidyI peptidase IV | DPP4 |
| Quercetin | Cytochrome P450 3A4 | CYP3A4 |
| Quercetin | Cytochrome P450 1A2 | CYP1A2 |
| Quercetin | Collagen alpha-1(III) chain | COL3A1 |
| Quercetin | Collagen alpha-1(1) chain | COL1A1 |
| Quercetin | Coagulation factor Xa | FXa |
| Quercetin | Coagulation factor VII | FVII |
| Quercetin | Cellular tumor antigen p53 | TP53 |
| Quercetin | Cell division control protein 2 homolog | CDC2 |
| Quercetin | C-C motif chemokine 2 | CCL2 |
| Quercetin | Cathepsin D | CTSD |
| Quercetin | Beta-2 adrenergic receptor | β2-AR |
| Quercetin | Aryl hydrocarbon receptor | AHR |
| Quercetin | Arachidonate 5-lipoxygenase | 5-LO |
| Quercetin | Apoptosis regulator BcI-2 | BCL2 |
| Quercetin | Androgen receptor | AR |
| Quercetin | Amine oxidase [flavin-containing] B | MAOB |
| Quercetin | Aldose reductase | ARI |
| Quercetin | Acetyl-CoA carboxylase 1 | ACCs |
| Quercetin | Acetylcholinesterase | AcCHS |
| Quercetin | 78 kDa glucose-regulated protein | GRP78 |
| Quercetin | 72 kDa type IV collagenase | TBE-1 |
| Sinapic acid | Prostaglandin G/H synthase 2 | PTGS2 |
| Sinapic acid | Prostaglandin G/H synthase 1 | PTGS1 |
| Sinapic acid | Nitric-oxide synthase, endothelial | eNOS |
| Sinapic acid | Nitric oxide synthase, inducible | iNOS |
| Sinapic acid | Glutamate receptor 2 | NR2B |
| Sinapic acid | Gamma-aminobutyric acid receptor subunit alpha-1 | GABAα1 |
| Sinapic acid | Choline O-acetyltransferase | CHAT |
| Sinapic acid | Beta-2 adrenergic receptor | β2-AR |
| Sinapic acid | Amine oxidase [flavin-containing] B | MAOB |
